# Pulmonary adiaspiromycosis in armadillos killed by motor vehicle collisions in Brazil

**DOI:** 10.1038/s41598-020-79521-6

**Published:** 2021-01-11

**Authors:** Pedro Enrique Navas-Suárez, Carlos Sacristán, Josue Díaz-Delgado, Débora R. Yogui, Mario Henrique Alves, Danny Fuentes-Castillo, Catalina Ospina-Pinto, Roberta Ramblas Zamana, Arnaud Leonard Jean Desbiez, Jose Luiz Catão-Dias

**Affiliations:** 1 Department of Pathology (VPT), School of Veterinary Medicine and Animal Science (FMVZ), University of São Paulo (USP), São Paulo, Brazil; 2 Texas A&M Veterinary Medical Diagnostic Laboratory, College Station, TX USA; 3 Instituto de Conservação de Animais Silvestres (ICAS), Campo Grande, Brazil; 4 Nashville Zoo, Nashville, USA; 5 Houston Zoo, Houston, USA; 6 Royal Zoological Society of Scotland, Edinburgh, UK

**Keywords:** Ecology, Zoology, Diseases, Pathogenesis

## Abstract

Knowledge of infectious diseases in wildlife provides important information for preventing potential outbreaks of zoonotic diseases. Adiaspiromycosis is a neglected human disease caused by dimorphic Onygenales fungi. The disease is produced by the inflammatory response against growing adiaspores, leading to granulomatous pneumonia. In humans, adiaspiromycosis is relevant in immunosuppressed patients. In animals, it is associated with pneumonia in fossorial species. Given the potential role of armadillos in the epidemiology of adiaspiromycosis, in this study, we sought to investigate the occurrence and pathological features of adiaspiromycosis in roadkilled armadillos. In total, 54 armadillo carcasses were suitable for postmortem pathologic examinations between February 2017 and 2020. Adiaspores, associated with granulomatous lesions, were observed in ten six-banded (*Euphractus sexcinctus*) and two southern naked-tailed armadillos (*Cabassous unicinctus*). A previously uncharacterized Onygenales species was molecularly identified in two *E. sexcinctus*. In summary, herein we report 12 cases of pulmonary adiaspiromycosis (PA) in two species of free-living armadillos in Brazil. Both, the morphology of the fungus, as well as the histopathological findings (granulomatous inflammatory response to adiaspores) are consistent with PA; however, as the molecular identification differs from the reported species, the potential impact of this fungus for human PA is unknown, and we cannot rule out its impact on public health.

## Introduction

Surveillance of infectious diseases in wildlife is a valuable tool for the prevention and reduction of human disease outbreaks^1,2^. Armadillos (order Cingulata) play a role in the epidemiological cycle of some zoonoses (e.g., hanseniasis and paracoccidioidomycosis)^3,4^. Despite being protected species, armadillo meat is commonly consumed in several South American countries^5,6^. Direct contact between humans and armadillos could pose a transmission route for several pathogens, thus, health investigations on armadillos may be relevant to public health.

Adiaspiromycosis is a fungal disease caused by the dimorphic fungi *Emmonsia crescens* and *Blastomyces parvus* (formerly known as *Emmonsia parva*) (family Ajellomycetaceae, order Onygenales), considered saprophytes and commonly isolated from the soil^7^. In contrast to other mycoses, once their conidia enter the host, mainly through respiratory via, they become adiaconidia (adiaspores), precluding their replication but increasing their size^8^. The disease is caused by the host´s immune response against the growing adiaspores, leading to the formation of granulomas^9^. The lesions are mainly restricted to the lungs and occasionally regional lymph nodes, although ocular and systemic adiaspiromycosis have also been described^8,10^. These lesions have been reported in humans, wild fossorial mammals (rodents, moles, armadillos), some species of carnivores, deer, horses and anurans (“Supplementary Material”).

The first description of the disease was made in wild rodents of the United States in the 1940s^11^. In humans, the first case was reported in France in the 1960s^12^. Adiaspiromycosis was widely studied in the 1970s, mainly in wild small mammals^13^. Nevertheless, reports have been intermittent during the last decades. While human cases are generally associated with immunosuppression, the clinico-pathological features in free-ranging animals remain largely unknown^14–18^. Human pulmonary adiaspiromycosis (PA) is generally an incidental finding in diagnostic images (X-rays and tomography) and can be a differential diagnosis of tuberculosis and/or pulmonary neoplasms; its definitive diagnosis is made by fungal culture and biopsy^19–21^. In Brazil, human cases have increased, and adiaspiromycosis is listed as a class II infectious agent (moderate individual risk and limited risk to the community) by the Ministry of Health^22^. In Brazilian animals, *Emmonsia* sp. was detected by PCR in one nine-banded armadillo (*Dasypus novemcinctus*) killed by motor-vehicle collision (MVC) in Botucatu, São Paulo State, however, no anatomopathological data were reported^23^. Adiaspiromycosis has been also described in three other armadillo species: hairy (*Chaetophractus villosus*), pichi (*Zaedyus pichiy*) and seven-banded armadillo (*Dasypus septemcinctus*) from Argentina^24^.

Since there are reports of PA in Brazil, and the etiologic agent has already been reported in armadillos (preliminarily by molecular technique)^23^, we hypothesized a possible role of armadillos in the epidemiology of PA. Considering that the number of wild animals killed by MVC in Brazil is remarkable^25^, and the potential role of armadillos in the epidemiology of adiaspiromycosis, in this study, we sought to investigate the occurrence and pathological features of adiaspiromycosis in roadkilled armadillos. Here we report 12 cases of PA in two species of wild armadillos of Brazil: the six-banded armadillo (*Euphractus sexcinctus*) and the southern naked-tailed armadillo (*Cabassous unicinctus*).

## Results

**Figure 1:**
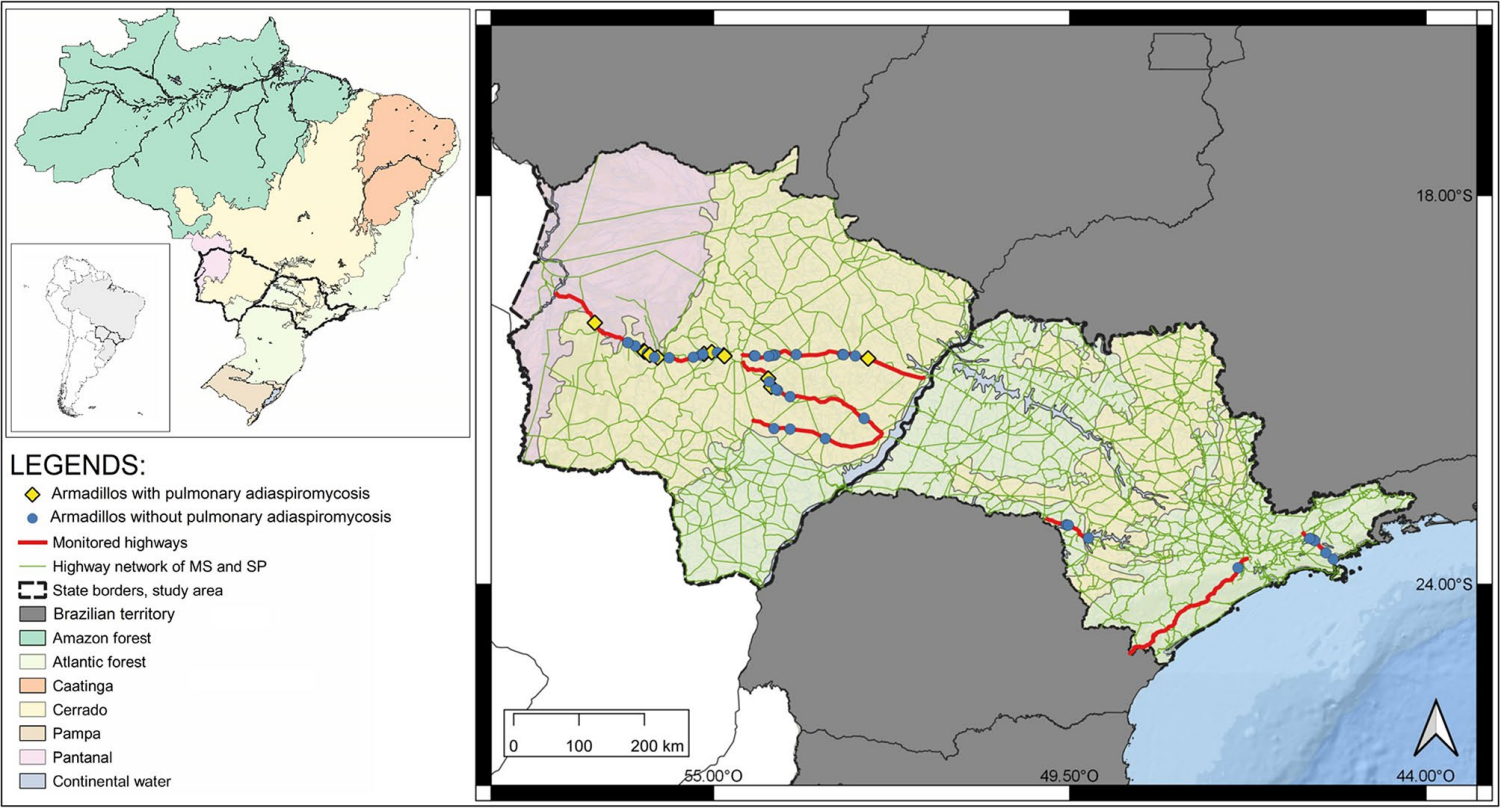
Geographic distribution of necropsied armadillos in roads of Mato Grosso do Sul and Sao Paulo states (Brazil) between February 2017 and February 2020. The map was created by software QGIS 3.16 (https://qgis.org/).

During the study period, 54 necropsies were performed in four armadillo species: nine-banded armadillo (*Dasypus novemcinctus*, 48.1%; 26/54), six-banded (*E. sexcinctus*, 38.9%; 21/54), southern naked-tailed (*C. unicinctus*, 9.3%; 5/54) and giant-armadillo (*Priodontes maximus*, 3.7%; 2/54). Geographic distribution of all armadillos is shown in Fig. 1.

**Figure 2:**
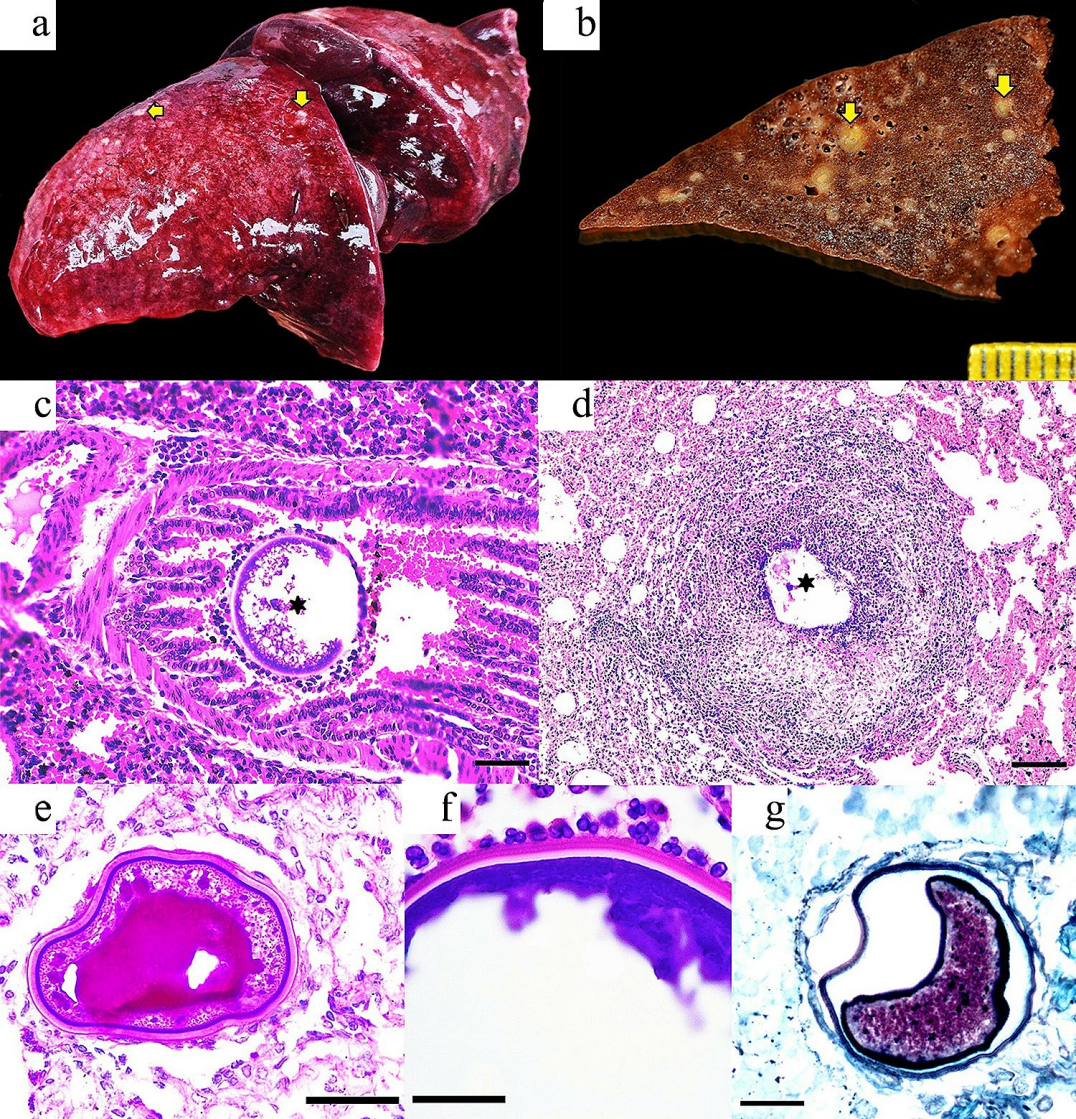
(**a**) Case 10; adult, female *E. sexcinctus*. The left lung lobes have heterogeneous red coloration as well as multifocal, pale tan to white subpleural nodules (yellow arrows). (**b**) Case 1; adult *E. sexcinctus*. The pulmonary parenchyma has multifocal subpleural and intraparenchymal nodules (yellow arrows) variably associated with bronchi or bronchioles. (**c**) Case 4; adult, male *Cabassous unicinctus*. Lungs. Note one adiaspores (asterisk) in the bronchial lumen surrounded by inflammatory cells. H&E; bar = 100 µm. (**d**) Case 10; Adult, female *E. sexcinctus*. Note a granuloma with fragments of the adiaspores wall (asterisk) in the necrotic center. H&E; bar = 200 µm. (**e**) Case 1; adult *E. sexcinctus*. Note the trilaminar wall of the adiaspores surrounded by few leukocytes. PAS; bar = 50 µm. (**f**) Case 1; adult *E. sexcinctus*. Detail of primarily neutrophils targeting the adiaspores wall. H&E; bar = 25 µm. (**g**) Case 1; adult *E. sexcinctus*. GMS stain highlights the adiaspores wall. Bar = 25 µm.

Ten *E. sexcinctus* (47.6%; 10/21) and two *C. unicinctus* (40%; 2/5) showed pulmonary histopathological findings with adiaspores morphologically most compatible with *Emmonsia crescens* or *Blastomyces parvus*. These cases were distributed in seven municipalities of MS, while no cases were found in SP. Complete biological and epidemiological data of armadillos with adiaspiromycosis are listed in Table 1. The main pulmonary gross findings associated with motor vehicle collisions (MVC) were hemorrhage (100%; 12/12), parenchymal rupture (66.7%; 8/12) and congestion (50%; 6/12) (“Supplementary Material”). Additionally, scattered 0.5–1 mm in-diameter, slightly demarcated, pale tan to yellow nodules were observed throughout the parenchyma of all lung lobes (25%; 3/12), these nodules corresponded to PA (Fig. 2).

**Table 1:** Biological and epidemiological data wild armadillos with pulmonary adiaspiromycosis died by motor vehicle collisions in Brazil, 2017–2020.

Case	Common name	Species	Age-class	Sex	Year	Month	Season	Municipality	State
1	SBA	*Euphractus sexcinctus*	Adult	Undetermined	2017	November	Rain	Miranda	MS
2	SBA	*Euphractus sexcinctus*	Adult	Female	2017	March	Rain	Terenos	MS
3	SBA	*Euphractus sexcinctus*	Adult	Female	2017	June	Dry	Terenos	MS
4	SNTA	*Cabassous unicinctus*	Adult	Male	2017	October	Rain	Água Clara	MS
5	SBA	*Euphractus sexcinctus*	Adult	Male	2017	November	Rain	Terenos	MS
6	SNTA	*Cabassous unicinctus*	Juvenile	Male	2017	December	Rain	Campo Grande	MS
7	SBA	*Euphractus sexcinctus*	Adult	Female	2017	December	Rain	Aquidauana	MS
8	SBA	*Euphractus sexcinctus*	Adult	Male	2018	January	Rain	Aquidauana	MS
9	SBA	*Euphractus sexcinctus*	Adult	Male	2018	February	Rain	Anastácio	MS
10	SBA	*Euphractus sexcinctus*	Adult	Female	2018	June	Dry	Anastácio	MS
11	SBA	*Euphractus sexcinctus*	Adult	Male	2018	July	Dry	Terenos	MS
12	SBA	*Euphractus sexcinctus*	Adult	Male	2018	July	Dry	Ribas do Rio Pardo	MS

*SBA* Six-Banded Armadillo, *SNTA* Southern Naked-Tailed Armadillo.

Microscopically, all these armadillos had pulmonary adiaspores with varying degrees of localized inflammation, ranging from none to marked granulomatous response (Table 2). Morphologically, adiaspores presented a bi- or trilaminar wall comprising a thin and brightly eosinophilic outer layer, a thick pale eosinophilic mid layer, and an internal basophilic layer which surrounded a core of round basophilic granular material but did not exhibit endospores. Adiaspores averaged 91.1 μm in diameter (ranged from 10.9 to 839.5 μm) and were distributed multifocally within the alveolar and bronchial interstitium spaces. Adiaspores’ wall components were highlighted by PAS, GMS and WS stains (Fig. 2). Inflammation severity correlated to adiaspores’ size and varied from few reactive macrophages surrounding the adiaspores to severe nodular granulomatous inflammation with large numbers of epithelioid macrophages, neutrophils, lymphocytes, eosinophils, rare multinucleated giant cells (foreign body type), and fibroplasia. The granulomas could contain a viable adiaspores (up to 200 μm in diameter) or cores with empty adiaspores admixed with neutrophils and necrotic cell debris. Several cases (91.7%, 11/12) had hyperplasia of the lymphoid tissue associated with bronchi and bronchioles. This prompted histochemical investigation for potential *Mycoplasma* sp. and/or *Filobacterium* sp. (formerly CAR Bacillus) structures; however, these were not detected by WS stain. One armadillo had pulmonary coinfection with a trichurid nematode leading to marked bronchitis. Complete microscopic findings are listed in Table 3. No fungal structures and/or associated inflammatory response were observed in other tissues {skin, skeletal muscle, tongue, oropharynx, tonsil, salivary glands, esophagus, stomach, small and large intestines, liver, gallbladder, pancreas, larynx, trachea, lung, heart, great vessels, thymus, spleen, lymph nodes (mandibular, prescapular, mediastinal and mesenteric), kidney, urinary bladder, thyroid and parathyroid glands, adrenal glands, diaphragm, cerebrum, cerebellum, eye, mammary gland, testicle, ovary, uterus, epididymis and prostate} examined.

**Table 2:** Morphological characteristics of the adiaspores observed in lungs of wild armadillos died by motor vehicle collisions in Brazil, 2017–2020.

Case	Number of adiaspores	Histologic distribution	Diameter (microns)	Min (microns)	Max (microns)	Inflammatory response
1	47	Alveolar, peribronchial	41.9	10.9	177.5	Granuloma
2	2	Peribronchial	66.1	52.3	79.9	Histiocytic infiltration
3	15	Alveolar, peribronchial	114.4	50.6	507.5	Histiocytic infiltration
4	3	Peribronchial, intrabronchial	292.4	257.7	319.8	Granuloma
5	10	Alveolar, peribronchial	39.0	16.9	81.5	Granuloma
6	1	Alveolar	277.9	277.9	277.9	Histiocytic infiltration
7	6	Alveolar, peribronchial	36.6	29.5	41.3	Histiocytic infiltration
8	1	Peribronchial	53.3	53.3	53.3	Histiocytic infiltration
9	4	Alveolar, peribronchial	79.5	69.4	94.7	Histiocytic infiltration
10	13	Alveolar, peribronchial	187.8	67.3	839.5	Granuloma
11	4	Peribronchial	95.6	34.8	220.9	Granuloma
12	5	Alveolar, intrabronchial	266.0	65.8	485.8	Granuloma

**Table 3:** Microscopic pulmonary findings in armadillos with pulmonary adiaspiromycosis died by motor vehicle collisions in Brazil, 2017–2020.

Microscopic finding	Total
**Vascular/hemodynamic**	
Congestion	100% (12/12)
Hemorrhage	100% (12/12)
Edema	83.3% (10/12)
Endothelial hypertrophy	75% (9/12)
Hemosiderosis	58.3% (7/12)
Tunica media hypertrophy/hyperplasia	50% (6/12)
Vasculitis/perivasculitis	50% (6/12)
**Alveoli, bronchial/bronchiolar submucosa, interstitium, pleura**	
Macrophagic infiltration^a^	100% (12/12)
Presence of adiaspores^a^	100% (12/12)
Lymphocytic infiltration	91.7% (11/12)
BALT hyperplasia	91.7% (11/12)
Hemorrhage	83.3% (10/12)
Atelectasia	83.3% (10/12)
Plasma cell infiltration	66.7% (8/12)
Bronchoconstriction	58.3% (7/12)
Granuloma	50% (6/12)
Anthracosis	50% (6/12)
Neutrophilic infiltration^a^	41.7% (5/12)
Eosinophilic infiltration	41.7% (5/12)
Hemosiderophages	41.7% (5/12)
Multinucleated giant cells	33.3% (4/12)
Nematode eggs (*Capillaria* sp.)	25% (3/12)
Mucus	25% (3/12)
Fibrin	16.7% (2/12)
Fibrosis	16.7% (2/12)
Necrotic cell debris	8.3% (1/12)
Aspirated particles	8.3% (1/12)
**Mucosa/epithelium**	
Sloughing/loss	100% (12/12)
Erosion	100% (12/12)
Hyperplasia of goblet cells	50% (6/12)
Bone marrow metaplasia	41.7% (5/12)
Multinucleated giant cells	33.3% (4/12)
Necrosis	25% (3/12)
Type II pneumocyte hyperplasia	25% (3/12)
Calcification	8.3% (1/12)

^a^Findings associated with PA.

*BALT* bronchi- and bronchiole-associated lymphoid tissue.

Two 640-bp sequences (after excluding primers) of the fragment comprised between 18S rRNA gene and 26S rRNA gene were obtained from frozen lung samples of two six-banded armadillos (cases 10 and 12) with pulmonary intralesional adiaspores. The 26S rRNA was also amplified and sequenced in these two animals. These sequences are available through the GenBank database [MT258564, MT258563, MT258566 and MT258565]. No sequences were recovered from the DNA extractions from FFPE lung samples of the remaining ten armadillos with adiaspores for any of the two selected PCR protocols.

**Figure 3:**
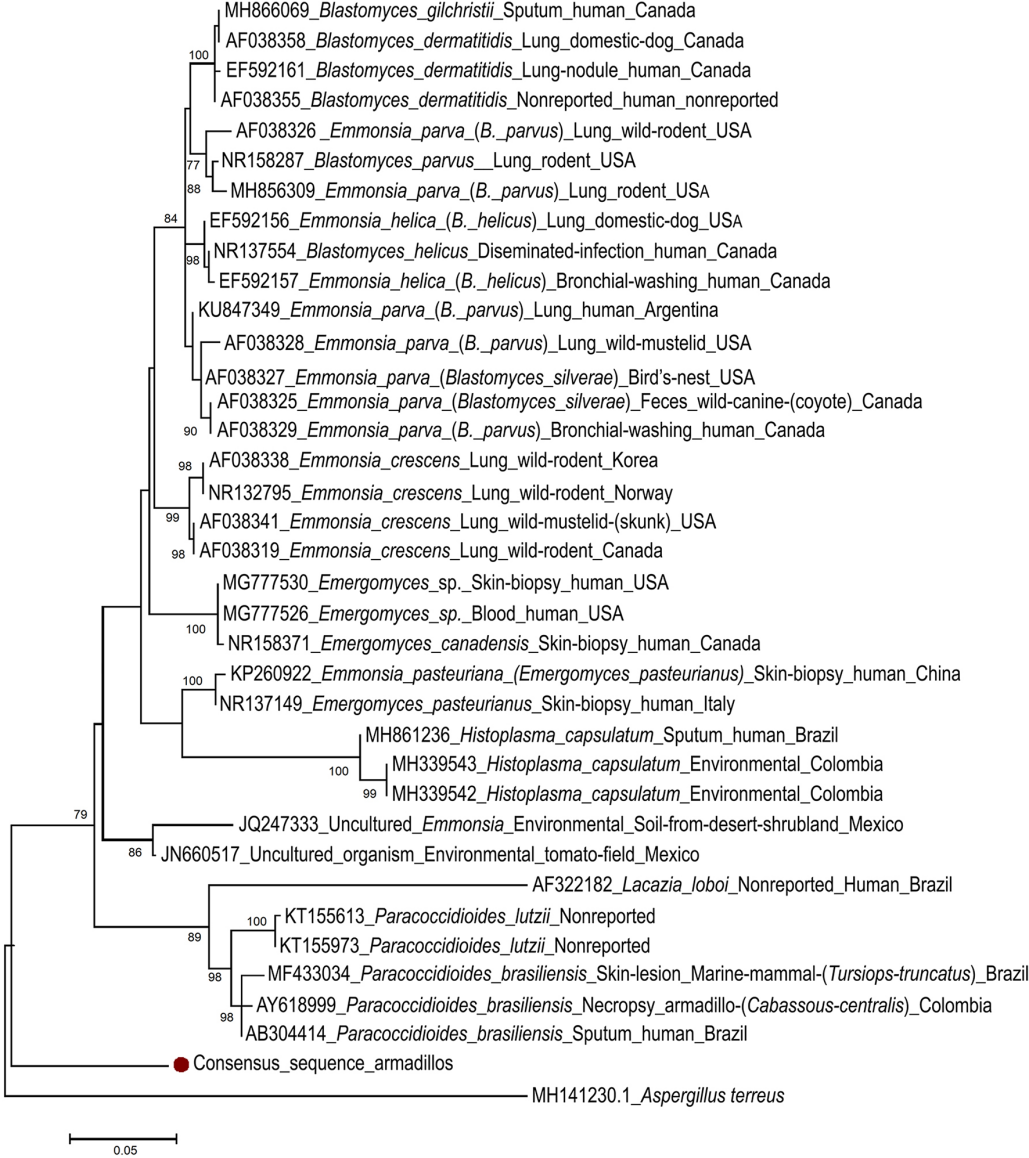
Maximum likelihood phylogram with 1000 bootstrap replicates of the alignment of the consensus internal transcriber spacer (ITS) 1, 5.8S rRNA gene and ITS 2 nucleotide sequence obtained in two six-banded armadillos (*Euphractus sexcinctus*) from this study, 35 selected fungi of the order Onygenales and *Aspergillus terreus* (MH141230) as outgroup.

The ITS sequences obtained from two six-banded armadillos (case 10 and 12) were identical between them, and had the highest nucleotide (NT) identity (87.6%) to an uncultured organism amplified from the soil of the rhizosphere of tomato (*Solanum lycopersicum*) in Mexico (JN660517), likely *E. crescens* (Cordero-Ramirez et al. 2012), followed by 86.5% similarity with two *E. crescens* sequences from lung samples of mammals (AF038334, AF038337). The unique sequence type found in armadillos clusters separately in our ITS phylogram (Fig. 3).

The two 26S rRNA sequences obtained in this study were highly similar (97.9% nt identity) to *Emmonsiellopsis terrestris* from the United States (AF038320) and Spain (KP101583), both identified from soil. The former sequence is identified in GenBank as *Emmonsiella* sp., but after that the same authors reclassified the sequence as *Emmonsiellopsis terrestris* (Marin-Felix et al. 2015). High similarity (97.6%) was also observed to strains of *Paracoccidioides brasiliensis* (U93304.1, AF038360.1), and to *Emergomyces europaeus* (EF592164.1), *E`. pasteurianus* (EF592152) and different *B. parvus* sequences (identified in GenBank as *E. parva*, e.g., AF038331.1, AF038329.1, AF038328.1) with 97.4% identity (Fig. 3).

## Discussion

Armadillos are among the most common species recorded in Brazilian roadkill monitoring programs, in this study were registered a total of 2941 armadillo carcasses over a 3-year period. This is primarily because some species in this group are generalist with large distribution areas and relatively high densities^26–28^. Examination of roadkill wildlife is pivotal for a better understanding and monitoring of infectious diseases with zoonotic potential, particularly in synanthropic and peri-urban wild animals^1,29^. In this study, armadillos killed by MVC were regarded as an important source of information for the surveillance of PA.

Adiaspiromycosis and emmonsiosis-like fungi are isolated from the soil^7^. Previous studies detailed that this order (Cingulata) presents a higher occurrence of infection by *Emmonsia* adiaspores when compared to other mammals (marsupials, rodents, lagomorphs and carnivores)^13^. It is reasonable to believe that the fossorial and digging habits of armadillos likely promote inhalation of soil adiaspores and subsequent lung lesions. However, we only found cases in two of the four species collected. However, the habits of *D. novemcintus* are less fossorial than *E. sexcinctus* and *C. unicinctus* is actually considered subterranean^30^. The low sample size of *P. maximus* (N = 2) may be why no cases were detected.

Microscopically, PA in man and animals is characterized by granulomatous pneumonia with intralesional adiaspores. The local inflammatory response includes histiocytes, neutrophils, epithelioid macrophages, lymphocytes, and occasionally multinucleated giant cells; and this is directly proportional to the adiaspores size. Adiaspores are usually bi- to trilaminar and their wall stains positive by PAS and GMS. *E. crescens* has a larger diameter (200–700 µm) compared to *B. parvus* (20–40 µm)^13,16,31^. These characteristics were observed in the armadillos analyzed in this study, however, even though the diameter of the adiaspores averaged 129.2 µm (Min = 10.9 µm; Max = 839.5 µm), being compatible with *E. crescens*, only 86.5% nt similarity for ITS region was obtained. The culture of these fungi was not performed due to their difficulty and the need of high biosecurity levels, fact that limited technically the morphologic characterization of the fungi^31^.

The identities of our ITS sequences with the nearest ones is even lower than those reported for core genes of *E. parva* isolates (88.6%), a species proposed as polyphyletic, and that those proposed for *E. crescens* strains (91.8%) based on genome identity^32^. The D1-D2 sequences are highly similar to other Onygenales species. Based on the molecular results, we identified a hitherto undescribed Onygenales species in pulmonary lesions of two six-banded armadillos causing adiaspiromycosis. Interestingly, aside from *E. crescens* and *B. parvus*, potential novel fungal species have been described in wild mammals with adiaspiromycosis, including the report of a unique sequence type more related to *Emmonsiellopsis* in two northern hairy-nosed wombats (*Lasiorhinus krefftii*) a fossorial species from Australia^13^. The zoonotic potential of the fungus infecting armadillos is unknown. Our molecular results raised the question about which adiaspore-producing species are infecting humans in Brazil, in light of most of the human cases are diagnosed only based on morphology, without ancillary molecular testing. To this date, in a literature review, at least 13 reports of human PA are available in the country (“Supplementary Material”). Furthermore, superficial adiaspiromycosis was reported to cause granulomatous conjunctivitis in children in the Amazon basin, and histopathologic examination of ocular nodules identified adiaspores-like structures^10^. Future studies molecularly identifying the fungal species in humans are necessary to explore the epidemiology of the disease, including the use of armadillo species as indicator of potential exposure areas to humans.

In summary, we provide pathological and molecular evidence of a relatively poorly known fungal disease in wild armadillos. Both, the morphology of the fungus, as well as the histopathological findings (granulomatous inflammatory response to adiaspores) are consistent with PA; however, as molecular identification differs from the reported species, the potential impact of this fungus for human PA is unknown, and we cannot rule out its impact on public health. To the authors’ knowledge this is the largest report of this disease in wild mammals from Brazil.

## Methods

### Road monitoring

Between February 2017 and February 2020, a periodic road monitoring was carried out to identify the number of armadillos killed by MVC in two states of Brazil, Mato Grosso do Sul (MS) and Sao Paulo (SP) (See *25* for details)^25^. Epidemiological and biological data [species, age class, sex, season (rainy or dry) and coordinates] were recorded from all carcasses. Roadkilled armadillos in good preservation status were selected for investigate the occurrence and pathological features of adiaspiromycosis by anatomopathological study^33^.

### Pulmonary adiaspiromycosis survey

Representative tissue samples of lungs, trachea and tracheobronchial lymph nodes were collected, and fixed in 10% neutral buffered formalin or frozen at − 20 °C or − 80 °C. These tissues were processed routinely, embedded on paraffin-wax, sectioned at 5 μm-thick and stained with hematoxylin and eosin (H&E) for routine microscopic analysis. Special stains periodic acid-Schiff (PAS), Grocott methenamine silver (GMS), Masson’s trichrome (MS) and Warthin–Starry stain (WS) were applied in selected cases to better characterize the histopathologic findings. Microphotographs of the adiaspores were collected and subsequently measured using the Image J software^34^.

### Molecular analysis

When adiaspores and/or associated lesions were found in the histopathological evaluation, total DNA was extracted from available frozen lung samples (n = 2) using the ZR Fungal/Bacterial DNA Miniprep kit (Zymo, Irvine, CA, USA), according to the manufacturer’s instructions. In cases, without available frozen sample (n = 10), DNA extraction of formalin-fixed and paraffin-embedded (FFPE) tissue was performed according to standardized protocols^35^. A panfungal PCR using the primers ITS1-F and ITS-4 at a melting temperature of 55 °C was performed to amplify a 700-bp fragment comprising the 18S rRNA gene, internal transcribed spacer-1 (ITS-1), 5.8S rRNA gene and ITS-2, until the 26S rRNA gene^36^. The variable regions D1 and D2 of the 26S rRNA gene of fungi were amplified by PCR using primers NL1 and NL4^37^. Amplicons of the expected size were purified with Exo-Sap IT (GE Healthcare, Waukesha, WI, USA) or Illustra DNA and Gel Band Purification kit (GE Healthcare, UK, if two bands were present in the agarose gel) and confirmed by direct Sanger sequencing. After a ClustalW alignment in Mega 7.0^38^, the obtained sequences (excluding primers) were compared with those available in GenBank using Blast search (http://www.ncbi.nlm.nih.gov/blast). The percentage of nucleotide (nt) identity of the obtained sequences to the closest available on GenBank was calculated based on p-distance ([1 − p-distance] × 100). A 1000 bootstrap maximum likelihood phylogram was constructed with the obtained ITS sequences, those from selected Onygenales species retrieved from GenBank and *Aspergillus terreus* as outgroup (MH141230).

### Ethical standards

This study was carried out in compliance with the System Authorization and Information on Biodiversity (SISBIO) of the Brazilian Institute of Environment and Renewable Natural Resources (IBAMA) (license number: 58745) and was approved by the Ethics Committee on Animal Use (CEUA) of the School of Veterinary Medicine and Animal Science—University of São Paulo (FMVZ-USP) (protocol number: 7198020317). All animals were dead at the time of necropsy; no animals were euthanized in this study.

## Supplementary Information

Supplementary Information.

## Data Availability

All data generated or analyzed during this study are included in this published article.
