# Author Correction: Pulmonary adiaspiromycosis in armadillos killed by motor vehicle collisions in Brazil

**DOI:** 10.1038/s41598-021-91343-8

**Published:** 2021-06-08

**Authors:** Pedro Enrique Navas‑Suárez, Carlos Sacristán, Josue Díaz‑Delgado, Débora R. Yogui, Mario Henrique Alves, Danny Fuentes‑Castillo, Catalina Ospina‑Pinto, Roberta Ramblas Zamana, Arnaud Leonard Jean Desbiez, Jose Luiz Catão‑Dias

**Affiliations:** 1 Department of Pathology (VPT), School of Veterinary Medicine and Animal Science (FMVZ), University of São Paulo (USP), São Paulo, Brazil; 2 Texas A&M Veterinary Medical Diagnostic Laboratory, College Station, TX USA; 3 Instituto de Conservação de Animais Silvestres (ICAS), Campo Grande, Brazil; 4 Nashville Zoo, Nashville, USA; 5 Houston Zoo, Houston, USA; 6 Royal Zoological Society of Scotland, Edinburgh, UK

Correction to: *Scientific Reports* 10.1038/s41598-020-79521-6, published online 11 January 2021

The original version of this Article contained an error in Reference 36, which was incorrectly given as:

Sacristán, C. *et al. *Identification of novel gammaherpesviruses in a South American fur seal (*Arctocephalus australis*) with ulcerative skin lesions. *J. Wildl. Dis.* **54**(3):592–596 (2018).

The correct reference is listed below:

Sacristán, C., Esperón, F., Ewbank, A.C., Kolesnikovas, C.K.M., Catão-Dias, J.L. Paracoccidioidomycosis ceti in an Atlantic bottlenose dolphin (*Tursiops truncatus*), Brazil. *Transbound. Emerg. Dis.* **65**(2):585–87 (2018).

The original Article has been corrected.

