# Supplementary material for: Pulmonary adiaspiromycosis in armadillos killed by motor vehicle collisions in Brazil
Source: Sci Rep. 2021 Jan 11;11:272. doi: 10.1038/s41598-020-79521-6 (PMC7801722; doi:10.1038/s41598-020-79521-6)
Supplement: Supplementary file 1 — Supplementary Information. [file 41598_2020_79521_MOESM1_ESM.pdf]

Pulmonary adiaspiromycosis in armadillos killed by motor vehicle collisions in Brazil.

Pedro Enrique Navas-Suárez[1]*, Carlos Sacristán[1], Josue Díaz-Delgado[1,2], Débora R. Yogui[3,4], Mario Henrique Alves[3,5], Danny Fuentes-Castillo[1], Catalina Ospina-Pinto[1], Roberta Ramblas Zamana[1], Arnaud Leonard Jean Desbiez[3,6], Jose Luiz Catão-Dias[1].

Supplementary Table 1. Historical and general information of human and animal cases of pulmonary adiaspiromycosis.

Year	Country	Species	Species fungi	Tissue	Samples	Diagnostic techniques	Macroscopic lesions	Micro Lesions	DFA	Condition	Occurrence	Reference
Human												
2013	Argentina	Human	*Emmonsia crescens*	Lung	Biopsy	Microscopy (HE, PAS, Groccot), Molecular (18S, 28S)	Micronodules in RX	Adiaspores were surrounded by a granulomatous inflammation	NI		100% (1/1)	1
1977	Brazil	Human	*Chrysosporium parvum var. Crescens*	Lung	Biopsy	Microscopy (HE, PAS, Groccot)	Multifocal nodular lesions	Adiaspores were surrounded by a severe granulomatous inflammation with high number of epithelioid macrophages	NI		100% (1/1)	2
1990	Brazil	Human	*Emmonsia crescens*	Lung	Biopsy	Microscopy (HE, PAS, Groccot)	Micronodules in RX	Adiaspores were surrounded by a severe granulomatous inflammation with high number of epithelioid macrophages	50-200		100% (2/2)	3
1992	Brazil	Human	*Chrysosporium sp.*	Lung	Necropsy	Microscopy (HE, PAS, Groccot), Ultraestructural	Multifocal nodular lesions	Adiaspores were surrounded by a granulomatous inflammation	380		100% (1/1)	4
1997	Brazil	Human	*Emmonsia parva var. crescens*	Lung	Biopsy	Microscopy (HE, PAS, Groccot)	Micronodules in RX	Adiaspores were surrounded by a granulomatous inflammation	NI		100% (3/3)	5
1997	Brazil	Human	*Emmonsia crescens*	Lungs	Biopsy	Microscopy (HE, PAS, Groccot), culture (sabouraud's Glucose agar)	Multifocal nodular lesions	Adiaspores were surrounded by a severe granulomatous inflammation with high number of epithelioid macrophages	NI		100% (1/1)	6
1998	Brazil	Human	*Emmonsia sp.*	Lung	Biopsy	Microscopy (HE, PAS, Groccot)	Multifocal nodular lesions	Adiaspores were surrounded by a severe granulomatous inflammation with high number of epithelioid macrophages	NI		100% (1/1)	7
2000	Brazil	Human	*Emmonsia parvum var. Crescens*	Lung	Biopsy	Microscopy (HE, PAS, Groccot)	Multifocal nodular lesions	Adiaspores were surrounded by a granulomatous inflammation	NI		100% (1/1)	2
2000	Brazil	Human	*Chrysosporium parvum var. Crescens*	Lung	Biopsy	Microscopy (HE, PAS, Groccot)	Multifocal nodular lesions	Adiaspores were surrounded by a severe granulomatous inflammation with high number of epithelioid macrophages	NI		100% (2/2)	8
2003	Brazil	Human	*Emmonsia crescens*	Mediastinic lymph node	Biopsy	Microscopy (HE, PAS, Groccot)	Multifocal nodular lesions	Adiaspores were surrounded by a granulomatous inflammation	400		100% (1/1)	9
2005	Brazil	Human	*Emmonsia sp.*	Eye	Biopsy	Microscopy (HE, PAS, Groccot)	Conjuntivitis	Adiaspores were surrounded by a granulomatous inflammation	NI		NI	10
2008	Brazil	Human	*Emmonsia parvum var. Crescens*	Lung	Biopsy	Microscopy (HE, PAS, Groccot)	Multifocal nodular lesions	Adiaspores were surrounded by a granulomatous inflammation	NI		100% (1/1)	11
2009	Brazil	Human	*Emmonsia sp.*	Lung	Biopsy	Microscopy (HE, PAS, Groccot), Molecular (18S, 28S)	Micronodules in RX	Adiaspores were surrounded by a granulomatous inflammation	NI		100% (1/1)	12
2015	China	Human	*Emmonsia pasteuriana*	Lung	Biopsy	Microscopy (HE, PAS, Groccot), Molecular (18S, 28S)	Micronodules in RX	Adiaspores were surrounded by a granulomatous inflammation	NI		100% (1/1)	13
1971	Czechoslovakia	Human	*Emmonsia crescens*	Lung	Biopsy	Microscopy (HE, PAS, Groccot)	Multifocal nodular lesions	Adiaspores were surrounded by a severe granulomatous inflammation with high number of epithelioid macrophages	250		100% (1/1)	14
1996	Finland	Human	*Emmonsia parva*	Lung	Biopsy	Microscopy (HE, PAS, Groccot)	Multifocal nodular lesions	Adiaspores were surrounded by a granulomatous inflammation	160-235		100% (1/1)	15
2008	France	Human	*Emmonsia crescens*	Lung	Biopsy	Microscopy (HE, PAS, Groccot), Molecular (18S, 28S)	Micronodules in RX	Empty granulomas	50-100		100% (1/1)	16
2003	Germany	Human	*Emmonsia crescens*	Lung	Biopsy	Microscopy (HE, PAS, Groccot), molecular (18S)	Multifocal nodular lesions	Adiaspores were surrounded by a granulomatous inflammation	NI		100% (1/1)	17
1979	India	Human	*Emmonsia crescens*	Skin	Biopsy	Microscopy (HE, PAS, Groccot)	Multifocal nodular lesions	Adiaspores were surrounded by a granulomatous inflammation	48-110		100% (2/2)	18
2012	India	Human	*Emmonsia pasteuriana*	Skin	Biopsy	Microscopy (HE, PAS, Groccot)	Multifocal nodular lesions	Adiaspores were surrounded by a severe granulomatous inflammation with high number of epithelioid macrophages	NI		100% (1/1)	19
1999	Israel	Human	*Emmonsia parva*	Lung	Biopsy	Microscopy (HE, PAS, Groccot), culture (sabouraud's Glucose agar)	Multifocal nodular lesions	Adiaspores were surrounded by a granulomatous inflammation	200-400		100% (1/1)	20
2010	Panama	Human	*Emmonsia crescens*	Lungs	Biopsy	Microscopy (HE, PAS, Groccot), culture (sabouraud's Glucose agar)	Multifocal nodular lesions	Adiaspores were surrounded by a severe granulomatous inflammation with high number of epithelioid macrophages	NI		100% (1/1)	21
2015	South Africa	Human	*Emmonsia sp.*	Lung	Biopsy	Microscopy (HE, PAS, Groccot), Molecular (18S, 28S)	Micronodules in RX	Adiaspores were surrounded by a granulomatous inflammation	NI		100% (1/1)	22
2008-2011	South Africa	Human	*Emmonsia pasteuriana*	Lung	Biopsy	Microscopy (HE, PAS, Groccot), Culture and Molecular	Multifocal nodular lesions	Adiaspores were surrounded by a granulomatous inflammation	NI		21% (13/62)	23

2007	Spain	Human	*Emmonsia crescens*	Lung	Necropsy	Microscopy (HE, PAS, Groccot), Molecular (ITS1, ITS2)	Multifocal nodular lesions	Adiaspores were surrounded by a granulomatous inflammation	NI		100% (1/1)	24
2011	Turkey	Human	*Emmonsia parva var. crescens*	Lung	Biopsy	Microscopy (HE, PAS, Groccot)	Micronodules in RX	Adiaspores were surrounded by a granulomatous inflammation	NI		100% (1/1)	25
2008	UK	Human	*Emmonsia crescens*	Lung	Biopsy	Microscopy (HE, PAS, Groccot)	Micronodules in RX	Subpleural granuloma contained a large fungal spore	NI		100% (1/1)	26
2015	UK	Human	*E pasteuriana*	Skin	Biopsy	Microscopy (HE, PAS, Groccot), molecular	Skin nodule	Adiaspores were surrounded by a granulomatous inflammation	NI		100% (1/1)	27
1998	USA	Human	*Emmonsia crescens*	Lung	Biopsy	Microscopy (HE, PAS, Groccot)	Micronodules in RX	Adiaspores were surrounded by a granulomatous inflammation	35-40		100% (1/1)	28
2004	USA	Human	*Chrysosporium sp.*	Skin	Biopsy	Microscopy (HE, PAS, Groccot)	Skin nodule	Adiaspores were surrounded by a granulomatous inflammation	NI		100% (1/1)	29
2011	USA	Human	*Emmonsia sp.*	Skin	Biopsy	Microscopy (HE, PAS, Groccot)	Skin nodule	Adiaspores were surrounded by a granulomatous inflammation	NI		100% (1/1)	30
2017	USA	Human	*Emmonsia sp.*	Lung	Biopsy	Microscopy (HE, PAS, Groccot)	Multifocal nodular lesions	Adiaspores were surrounded by a granulomatous inflammation	NI		100% (1/1)	31
1973	Venezuela	Human	*Chrysosporium sp.*	Lung	Necropsy	Microscopy (HE, PAS, Groccot), molecular (28S)	No gross lesions	Adiaspores were surrounded by a granulomatous inflammation	NI		100% (2/2)	32
Animals												
Rodents												
1962	Argentina	*Cavia pamparum*	*Emmonsia sp.*	Lung	Necropsy	Microscopy (HE, PAS, Groccot)	Multifocal nodular lesions	Adiaspores were surrounded by a granulomatous inflammation	NI	Free ranging	28.6& (2/7)	33
1962	Argentina	*Dolichotis patagonum*	*Emmonsia sp.*	Lung	Necropsy	Microscopy (HE, PAS, Groccot)	Multifocal nodular lesions	Adiaspores were surrounded by a granulomatous inflammation	NI	Free ranging	5.6% (1/18)	33
1962	Argentina	*Lagostomus maximus*	*Emmonsia sp.*	Lung	Necropsy	Microscopy (HE, PAS, Groccot)	Multifocal nodular lesions	Adiaspores were surrounded by a granulomatous inflammation	NI	Free ranging	14.3% (2/14)	33
1977	Austria	*Rodentia, not determined*	*Emmonsia crescens*	Lung	Necropsy	Microscopy (HE, PAS, Groccot), Culture (Sabhi agar)	Multifocal nodular lesions	Adiaspores were surrounded by a granulomatous inflammation	225-634	Free ranging	5.6% (10/177)	34
1960	Bulgary	*Apodemus agrarius*	*Emmonsia crescens*	Lung	Necropsy	Microscopy (HE, PAS, Groccot), culture (sabouraud's Glucose agar)	Multifocal nodular lesions	Adiaspores were surrounded by a granulomatous inflammation	NI	Free ranging	1% (4/400)	35
1960	Bulgary	*Apodemus flavicollis*	*Emmonsia crescens*	Lung	Necropsy	Microscopy (HE, PAS, Groccot), culture (sabouraud's Glucose agar)	Multifocal nodular lesions	Adiaspores were surrounded by a granulomatous inflammation	NI	Free ranging	5.8% (20/343)	35
1960	Bulgary	*Apodemus sylvaticus*	*Emmonsia crescens*	Lung	Necropsy	Microscopy (HE, PAS, Groccot), culture (sabouraud's Glucose agar)	Multifocal nodular lesions	Adiaspores were surrounded by a granulomatous inflammation	NI	Free ranging	3.6% (25/702)	35
1960	Bulgary	*Clethrionomys glareolus*	*Emmonsia crescens*	Lung	Necropsy	Microscopy (HE, PAS, Groccot), culture (sabouraud's Glucose agar)	Multifocal nodular lesions	Adiaspores were surrounded by a granulomatous inflammation	NI	Free ranging	24.6% (15/61)	35
1960	Bulgary	*Glis glis*	*Emmonsia crescens*	Lung	Necropsy	Microscopy (HE, PAS, Groccot), culture (sabouraud's Glucose agar)	Multifocal nodular lesions	Adiaspores were surrounded by a granulomatous inflammation	NI	Free ranging	40% (4/10)	35
1960	Bulgary	*Microtus arvalis*	*Emmonsia crescens*	Lung	Necropsy	Microscopy (HE, PAS, Groccot), culture (sabouraud's Glucose agar)	Multifocal nodular lesions	Adiaspores were surrounded by a granulomatous inflammation	NI	Free ranging	2.4% (4/165)	35
1960	Bulgary	*Mus musculus*	*Emmonsia crescens*	Lung	Necropsy	Microscopy (HE, PAS, Groccot), culture (sabouraud's Glucose agar)	Multifocal nodular lesions	Adiaspores were surrounded by a granulomatous inflammation	NI	Free ranging	1.2% (5/424)	35
1960	Bulgary	*Neomys anomalus*	*Emmonsia crescens*	Lung	Necropsy	Microscopy (HE, PAS, Groccot), culture (sabouraud's Glucose agar)	Multifocal nodular lesions	Adiaspores were surrounded by a granulomatous inflammation	NI	Free ranging	1.9% (5/263)	35
1960	Bulgary	*Neomys fodiens*	*Emmonsia crescens*	Lung	Necropsy	Microscopy (HE, PAS, Groccot), culture (sabouraud's Glucose agar)	Multifocal nodular lesions	Adiaspores were surrounded by a granulomatous inflammation	NI	Free ranging	2% (1/50)	35
1960	Bulgary	*Pitymys subterraneus*	*Emmonsia crescens*	Lung	Necropsy	Microscopy (HE, PAS, Groccot), culture (sabouraud's Glucose agar)	Multifocal nodular lesions	Adiaspores were surrounded by a granulomatous inflammation	NI	Free ranging	1.5% (1/68)	35
1960	Bulgary	*Rattus norvegicus*	*Emmonsia crescens*	Lung	Necropsy	Microscopy (HE, PAS, Groccot), culture (sabouraud's Glucose agar)	Multifocal nodular lesions	Adiaspores were surrounded by a granulomatous inflammation	NI	Free ranging	4.2% (1/24)	35
1960	Bulgary	*Rattus rattus*	*Emmonsia crescens*	Lung	Necropsy	Microscopy (HE, PAS, Groccot), culture (sabouraud's Glucose agar)	Multifocal nodular lesions	Adiaspores were surrounded by a granulomatous inflammation	NI	Free ranging	2.9% (2/70)	35
1957	Canada	*Wild rodents (Sorex, Microtus, Peromyscus)*	*Emmonsia sp.*	Lung	Necropsy	Microscopy (HE)		Adiaspores were surrounded by a granulomatous inflammation	NI	Free ranging	-	36
1969	Canada	*Ochotona princeps*	*Emmonsia crescens*	Lung	Necropsy	Microscopy (HE, PAS, Groccot), culture (sabouraud's Glucose agar)	Multifocal nodular lesions	Adiaspores were surrounded by a granulomatous inflammation	240	Free ranging	0.4% (1/240)	37
1970	Canada	*Citellus franklini*	*Emmonsia crescens*	Lung	Necropsy	Microscopy (HE, PAS, Groccot), culture (sabouraud's Glucose agar)	Multifocal nodular lesions	Adiaspores were surrounded by a granulomatous inflammation	NI	Free ranging	79.5% (35/44)	38
1970	Canada	*Citellus richardsoni*	*Emmonsia crescens*	Lung	Necropsy	Microscopy (HE, PAS, Groccot), culture (sabouraud's Glucose agar)	Multifocal nodular lesions	Adiaspores were surrounded by a granulomatous inflammation	NI	Free ranging	2.5% (2/81)	38
1970	Canada	*Citellus tridecemlineatus*	*Emmonsia crescens*	Lung	Necropsy	Microscopy (HE, PAS, Groccot), culture (sabouraud's Glucose agar)	Multifocal nodular lesions	Adiaspores were surrounded by a granulomatous inflammation	NI	Free ranging	17.6% (3/17)	38
1971	Canada	*Poliocitellus franklinii*	*Emmonsia crescens*	Lung	Necropsy	Microscopy (HE, PAS, Groccot), culture (sabouraud's Glucose agar)	Multifocal nodular lesions	Adiaspores were surrounded by a granulomatous inflammation	20-70	Free ranging	25% (3/12)	37
1974	Czechoslovakia	*Apodemus flavicollis*	*Emmonsia crescens*	Lung	Necropsy	Microscopy (HE, PAS, Groccot)	Multifocal nodular lesions	Adiaspores were surrounded by a severe granulomatous inflammation with high number of epithelioid macrophages	70-310	Experimental infection	77.8% (7/9)	39
1974	Czechoslovakia	*Apodemus sylvaticus*	*Emmonsia crescens*	Lung	Necropsy	Microscopy (HE, PAS, Groccot)	Multifocal nodular lesions	Adiaspores were surrounded by a severe granulomatous inflammation with high number of epithelioid macrophages	70-310	Experimental infection	22.2% (2/9)	39
1974	Czechoslovakia	*Clethrionomys glareolus*	*Emmonsia crescens*	Lung	Necropsy	Microscopy (HE, PAS, Groccot)	Multifocal nodular lesions	Adiaspores were surrounded by a severe granulomatous inflammation with high number of epithelioid macrophages	70-310	Experimental infection	66.7% (4/6)	39

1974	Czechoslovakia	*Microtus arvalis*	*Emmonsia crescens*	Lung	Necropsy	Microscopy (HE, PAS, Groccot)	Multifocal nodular lesions	Adiaspores were surrounded by a severe granulomatous inflammation with high number of epithelioid macrophages	70-310	Experimental infection	75.6% (34/45)	39
1974	Czechoslovakia	*Microtus agrestis*	*Emmonsia crescens*	Lung	Necropsy	Microscopy (HE, PAS, Groccot)	Multifocal nodular lesions	Adiaspores were surrounded by a severe granulomatous inflammation with high number of epithelioid macrophages	70-310	Experimental infection	57.1& (4/7)	39
1974	Czechoslovakia	*Mus musculus*	*Emmonsia crescens*	Lung	Necropsy	Microscopy (HE, PAS, Groccot)	Multifocal nodular lesions	Adiaspores were surrounded by a severe granulomatous inflammation with high number of epithelioid macrophages	70-310	Experimental infection	75% (3/4)	39
1986	Czechoslovakia	*Clethrionomys glareolus*	*Emmonsia crescens*	Lung	Necropsy	Microscopy (HE, PAS, Groccot), Culture (Sabhi agar)	Multifocal nodular lesions	Adiaspores were surrounded by a granulomatous inflammation	225-634	Free ranging	4.8% (1/21)	40
1986	Czechoslovakia	*Microtus agrestis*	*Emmonsia crescens*	Lung	Necropsy	Microscopy (HE, PAS, Groccot), Culture (Sabhi agar)	Multifocal nodular lesions	Adiaspores were surrounded by a granulomatous inflammation	225-634	Free ranging	8.4% (23/273)	40
1989	Czechoslovakia	*Apodemus flavicollis*	*Emmonsia parva var. crescens*	Lung	Necropsy	Microscopy (HE, PAS, Groccot), Culture (Sabhi agar)	Multifocal nodular lesions	Adiaspores were surrounded by a granulomatous inflammation	76-497	Free ranging	25.4% (48/189)	41
1989	Czechoslovakia	*Apodemus microps*	*Emmonsia parva var. crescens*	Lung	Necropsy	Microscopy (HE, PAS, Groccot), Culture (Sabhi agar)	Multifocal nodular lesions	Adiaspores were surrounded by a granulomatous inflammation	76-497	Free ranging	10.6% (17/160)	41
1989	Czechoslovakia	*Apodemus sylvaticus*	*Emmonsia parva var. crescens*	Lung	Necropsy	Microscopy (HE, PAS, Groccot), Culture (Sabhi agar)	Multifocal nodular lesions	Adiaspores were surrounded by a granulomatous inflammation	76-497	Free ranging	21.5% (29/135)	41
1989	Czechoslovakia	*Clethrionomys glareolus*	*Emmonsia parva var. crescens*	Lung	Necropsy	Microscopy (HE, PAS, Groccot), Culture (Sabhi agar)	Multifocal nodular lesions	Adiaspores were surrounded by a granulomatous inflammation	76-497	Free ranging	29.8% (48/161	41
1989	Czechoslovakia	*Microtus agrestis*	*Emmonsia parva var. crescens*	Lung	Necropsy	Microscopy (HE, PAS, Groccot), Culture (Sabhi agar)	Multifocal nodular lesions	Adiaspores were surrounded by a granulomatous inflammation	76-497	Free ranging	4.6% (14/307)	41
1990	Czechoslovakia	*Apodemus flavicollis*	*Emmonsia parva var. crescens*	Lung	Necropsy	Microscopy (HE, PAS, Groccot), Culture (Sabhi agar)	Multifocal nodular lesions	Adiaspores were surrounded by a granulomatous inflammation	NI	Free ranging	21.2% (75/353)	42
1990	Czechoslovakia	*Apodemus sylvaticus*	*Emmonsia parva var. crescens*	Lung	Necropsy	Microscopy (HE, PAS, Groccot), Culture (Sabhi agar)	Multifocal nodular lesions	Adiaspores were surrounded by a granulomatous inflammation	NI	Free ranging	21.2% (10/43)	42
1990	Czechoslovakia	*Clethrionomys glareolus*	*Emmonsia parva var. crescens*	Lung	Necropsy	Microscopy (HE, PAS, Groccot), Culture (Sabhi agar)	Multifocal nodular lesions	Adiaspores were surrounded by a granulomatous inflammation	NI	Free ranging	35.3% (65/184)	42
1998	Czechoslovakia	*Rodentia, not determined*	*Emmonsia parva var. crescens*	Lung	Necropsy	Microscopy (HE, PAS, Groccot), Culture (Sabhi agar)	Multifocal nodular lesions	Adiaspores were surrounded by a granulomatous inflammation	NI	Free ranging	8.2% (15/184)	43
1971-1975	Czechoslovakia	*Apodemus flavicollis*	*Emmonsia crescens*	Lung	Necropsy	Microscopy (HE, PAS, Groccot), culture (sabouraud's Glucose agar)	Multifocal nodular lesions	Adiaspores were surrounded by a granulomatous inflammation	30-600	Free ranging	4% (4/101)	44
1971-1975	Czechoslovakia	*Apodemus sylvaticus*	*Emmonsia crescens*	Lung	Necropsy	Microscopy (HE, PAS, Groccot), culture (sabouraud's Glucose agar)	Multifocal nodular lesions	Adiaspores were surrounded by a granulomatous inflammation	30-600	Free ranging	6.5% (3/46)	44
1971-1975	Czechoslovakia	*Clethrionomys glareolus*	*Emmonsia crescens*	Lung	Necropsy	Microscopy (HE, PAS, Groccot), culture (sabouraud's Glucose agar)	Multifocal nodular lesions	Adiaspores were surrounded by a granulomatous inflammation	30-600	Free ranging	12.5% (11/88)	44
1971-1975	Czechoslovakia	*Cricetus cricetus*	*Emmonsia crescens*	Lung	Necropsy	Microscopy (HE, PAS, Groccot), culture (sabouraud's Glucose agar)	Multifocal nodular lesions	Adiaspores were surrounded by a granulomatous inflammation	30-600	Free ranging	5.2% (8/153)	44
1971-1975	Czechoslovakia	*Dryomys nitedula*	*Emmonsia crescens*	Lung	Necropsy	Microscopy (HE, PAS, Groccot), culture (sabouraud's Glucose agar)	Multifocal nodular lesions	Adiaspores were surrounded by a granulomatous inflammation	30-600	Free ranging	15% (3/20)	44
1971-1975	Czechoslovakia	*Glis glis*	*Emmonsia crescens*	Lung	Necropsy	Microscopy (HE, PAS, Groccot), culture (sabouraud's Glucose agar)	Multifocal nodular lesions	Adiaspores were surrounded by a granulomatous inflammation	30-600	Free ranging	16.7% (3/18)	44
1971-1975	Czechoslovakia	*Microtus agrestis*	*Emmonsia crescens*	Lung	Necropsy	Microscopy (HE, PAS, Groccot), culture (sabouraud's Glucose agar)	Multifocal nodular lesions	Adiaspores were surrounded by a granulomatous inflammation	30-600	Free ranging	21.1% (4/19)	44
1971-1975	Czechoslovakia	*Microtus arvalis*	*Emmonsia crescens*	Lung	Necropsy	Microscopy (HE, PAS, Groccot), culture (sabouraud's Glucose agar)	Multifocal nodular lesions	Adiaspores were surrounded by a granulomatous inflammation	30-600	Free ranging	7.6% (17/223)	44
1971-1975	Czechoslovakia	*Ondatra zibethicus*	*Emmonsia crescens*	Lung	Necropsy	Microscopy (HE, PAS, Groccot), culture (sabouraud's Glucose agar)	Multifocal nodular lesions	Adiaspores were surrounded by a granulomatous inflammation	30-600	Free ranging	15% (17/113)	44
1971-1975	Czechoslovakia	*Sciurus vulgaris*	*Emmonsia crescens*	Lung	Necropsy	Microscopy (HE, PAS, Groccot), culture (sabouraud's Glucose agar)	Multifocal nodular lesions	Adiaspores were surrounded by a granulomatous inflammation	30-600	Free ranging	20.5% (66/321)	44
1986-1997	Czechoslovakia	*Apodemus flavicollis*	*Emmonsia crescens*	Lung	Necropsy	Microscopy (HE, PAS, Groccot), Culture (Sabhi agar)	Multifocal nodular lesions	Adiaspores were surrounded by a granulomatous inflammation	46-451	Free ranging	16% (357/2172)	45
1986-1997	Czechoslovakia	*Apodemus microps*	*Emmonsia crescens*	Lung	Necropsy	Microscopy (HE, PAS, Groccot), Culture (Sabhi agar)	Multifocal nodular lesions	Adiaspores were surrounded by a granulomatous inflammation	46-451	Free ranging	9% (23/265)	45
1986-1997	Czechoslovakia	*Apodemus sylvaticus*	*Emmonsia crescens*	Lung	Necropsy	Microscopy (HE, PAS, Groccot), Culture (Sabhi agar)	Multifocal nodular lesions	Adiaspores were surrounded by a granulomatous inflammation	46-451	Free ranging	11% (220/1981)	45
1986-1997	Czechoslovakia	*Arvicola terrestris*	*Emmonsia crescens*	Lung	Necropsy	Microscopy (HE, PAS, Groccot), Culture (Sabhi agar)	Multifocal nodular lesions	Adiaspores were surrounded by a granulomatous inflammation	46-451	Free ranging	17% (1/6)	45
1986-1997	Czechoslovakia	*Clethrionomys glareolus*	*Emmonsia crescens*	Lung	Necropsy	Microscopy (HE, PAS, Groccot), Culture (Sabhi agar)	Multifocal nodular lesions	Adiaspores were surrounded by a granulomatous inflammation	46-451	Free ranging	23% (441/1934)	45
1986-1997	Czechoslovakia	*Cricetus cricetus*	*Emmonsia crescens*	Lung	Necropsy	Microscopy (HE, PAS, Groccot), Culture (Sabhi agar)	Multifocal nodular lesions	Adiaspores were surrounded by a granulomatous inflammation	46-451	Free ranging	100% (1/1)	45
1986-1997	Czechoslovakia	*Microtus agrestis*	*Emmonsia crescens*	Lung	Necropsy	Microscopy (HE, PAS, Groccot), Culture (Sabhi agar)	Multifocal nodular lesions	Adiaspores were surrounded by a granulomatous inflammation	46-451	Free ranging	7% (98/1439)	45
1986-1997	Czechoslovakia	*Microtus arvalis*	*Emmonsia crescens*	Lung	Necropsy	Microscopy (HE, PAS, Groccot), Culture (Sabhi agar)	Multifocal nodular lesions	Adiaspores were surrounded by a granulomatous inflammation	46-451	Free ranging	7% (93/1275)	45
1986-1997	Czechoslovakia	*Microtus subterraneus*	*Emmonsia crescens*	Lung	Necropsy	Microscopy (HE, PAS, Groccot), Culture (Sabhi agar)	Multifocal nodular lesions	Adiaspores were surrounded by a granulomatous inflammation	46-451	Free ranging	14% (11/81)	45
1986-1997	Czechoslovakia	*Neomys fodiens*	*Emmonsia crescens*	Lung	Necropsy	Microscopy (HE, PAS, Groccot), Culture (Sabhi agar)	Multifocal nodular lesions	Adiaspores were surrounded by a granulomatous inflammation	46-451	Free ranging	5% (2/40)	45
1986-1997	Czechoslovakia	*Ondatra zibethicus*	*Emmonsia crescens*	Lung	Necropsy	Microscopy (HE, PAS, Groccot), Culture (Sabhi agar)	Multifocal nodular lesions	Adiaspores were surrounded by a granulomatous inflammation	46-451	Free ranging	33% (1/3)	45
1988-1993	Czechoslovakia	*Apodemus flavicollis*	*Emmonsia parva var. crescens*	Lung	Necropsy	Microscopy (HE, PAS, Groccot), Culture (Sabhi agar)	Multifocal nodular lesions	Adiaspores were surrounded by a granulomatous inflammation	NI	Free ranging	23.3% (102/437)	46

1988-1993	Czechoslovakia	*Apodemus sylvaticus*	*Emmonsia parva var. crescens*	Lung	Necropsy	Microscopy (HE, PAS, Groccot), Culture (Sabhi agar)	Multifocal nodular lesions	Adiaspores were surrounded by a granulomatous inflammation	NI	Free ranging	19.8% (59/298)	46
1988-1993	Czechoslovakia	*Clethrionomys glareolus*	*Emmonsia parva var. crescens*	Lung	Necropsy	Microscopy (HE, PAS, Groccot), Culture (Sabhi agar)	Multifocal nodular lesions	Adiaspores were surrounded by a granulomatous inflammation	NI	Free ranging	36.1% (84/233)	46
1988-1993	Czechoslovakia	*Microtus arvalis*	*Emmonsia parva var. crescens*	Lung	Necropsy	Microscopy (HE, PAS, Groccot), Culture (Sabhi agar)	Multifocal nodular lesions	Adiaspores were surrounded by a granulomatous inflammation	NI	Free ranging	19.6% (39/199)	46
1988-1993	Czechoslovakia	*Microtus subterraneus*	*Emmonsia parva var. crescens*	Lung	Necropsy	Microscopy (HE, PAS, Groccot), Culture (Sabhi agar)	Multifocal nodular lesions	Adiaspores were surrounded by a granulomatous inflammation	NI	Free ranging	33% (1/3)	46
1988-1993	Czechoslovakia	*Rodentia, not determined*	*Emmonsia parva var. crescens*	Lung	Necropsy	Microscopy (HE, PAS, Groccot), Culture (Sabhi agar)	Multifocal nodular lesions	Adiaspores were surrounded by a granulomatous inflammation	46-451	Free ranging	16.6% (190/1143)	47
1999-2000	Czechoslovakia	*Apodemus flavicollis*	*Emmonsia parva var. crescens*	Lung	Necropsy	Microscopy (HE, PAS, Groccot)	Multifocal nodular lesions	Adiaspores were surrounded by a granulomatous inflammation	83-575	Free ranging	16.7% (4/24)	48
1999-2000	Czechoslovakia	*Apodemus sylvaticus*	*Emmonsia parva var. crescens*	Lung	Necropsy	Microscopy (HE, PAS, Groccot)	Multifocal nodular lesions	Adiaspores were surrounded by a granulomatous inflammation	83-575	Free ranging	3.2% (1/31)	48
1999-2000	Czechoslovakia	*Clethrionomys glareolus*	*Emmonsia parva var. crescens*	Lung	Necropsy	Microscopy (HE, PAS, Groccot)	Multifocal nodular lesions	Adiaspores were surrounded by a granulomatous inflammation	83-575	Free ranging	15.6% (7/45)	48
1999-2000	Czechoslovakia	*Mus musculus*	*Emmonsia parva var. crescens*	Lung	Necropsy	Microscopy (HE, PAS, Groccot)	Multifocal nodular lesions	Adiaspores were surrounded by a granulomatous inflammation	83-575	Free ranging	5.6% (1/18)	48
2001	Israel	*Spalax galili*	*Emmonsia parva var. crescens*	Lung	Necropsy	Microscopy (HE, PAS, Groccot), Culture (Sabhi agar)	Multifocal nodular lesions	Adiaspores were surrounded by a granulomatous inflammation	17-192	Free ranging	28% (5/18)	49
2010	Italy	*Hystrix cristata*	*Emmonsia crescens*	Lung	Necropsy	Microscopy (HE, PAS, Groccot), Molecular (ITS1)	Multifocal nodular lesions	Adiaspores were surrounded by a severe granulomatous inflammation with high number of epithelioid macrophages	150-450	Free ranging	100% (1/1)	50
1971	Japan	*Apodemus argenteus*	*Emmonsia crescens*	Lung	Necropsy	Microscopy (HE, PAS, Groccot)	Multifocal nodular lesions	Adiaspores were surrounded by a granulomatous inflammation	544	Free ranging	2% (1/50)	51
1972	Japan	*Ochotona hyperborea yesoensis kishida*	*Chrysosporium parvum var crescens*	Lung	Necropsy	Microscopy (HE, PAS, Groccot)	Multifocal nodular lesions	Adiaspores were surrounded by a granulomatous inflammation	300-420	Free ranging	100% (2/2)	52
2017	Japan	*Rattus norvegicus*	*Emmonsia crescens*	Lung	Necropsy	Microscopy (HE, PAS, Groccot)	Multifocal nodular lesions	Adiaspores were surrounded by a granulomatous inflammation	250-350	Free ranging	100% (1/1)	53
2017	Japan	*Sciurus vulgaris orientis*	*Emmonsia crescens*	Lung	Necropsy	Microscopy (HE, PAS, Groccot)	Multifocal nodular lesions	Adiaspores were surrounded by a granulomatous inflammation	250-350	Free ranging	100% (1/1)	53
2012	Korea	*Apodemus agrarius*	*Emmonsia sp.*	Lung	Necropsy	Microscopy (HE, PAS, Groccot)	Multifocal nodular lesions	Adiaspores were surrounded by a granulomatous inflammation	195-500	Free ranging	100% (1/1)	54
2017	Poland	*Castor fiber*	*Emmonsia sp.*	Lung	Necropsy	Microscopy (HE, PAS, Groccot)		Adiaspores were surrounded by a granulomatous inflammation	160-430	Free ranging	8% (2/25)	55
1960	Sweden	*Apodemus flavicollis*	*Emmonsia parva*	Lung	Necropsy	Microscopy (HE, PAS, Groccot), culture (sabouraud's Glucose agar)	Multifocal nodular lesions	Adiaspores were surrounded by a granulomatous inflammation	NI	Free ranging	6.6% (4/61)	56
1960	Sweden	*Apodemus sylvaticus*	*Emmonsia parva*	Lung	Necropsy	Microscopy (HE, PAS, Groccot), culture (sabouraud's Glucose agar)	Multifocal nodular lesions	Adiaspores were surrounded by a granulomatous inflammation	NI	Free ranging	2.9% (1/34)	56
1960	Sweden	*Clethrionomys glareolus*	*Emmonsia parva*	Lung	Necropsy	Microscopy (HE, PAS, Groccot), culture (sabouraud's Glucose agar)	Multifocal nodular lesions	Adiaspores were surrounded by a granulomatous inflammation	NI	Free ranging	10% (1/10)	56
1999	Sweden	*Castor fiber*	*Emmonsia parvum var. Crescens*	Lung	Necropsy	Microscopy (HE, PAS, Groccot)	Multifocal nodular lesions	Adiaspores were surrounded by a severe granulomatous inflammation with high number of epithelioid macrophages	100-200	Free ranging	100% (1/1)	57
2003	UK	*Arvicola terrestris*	*Emmonsia crescens*	Lung	Necropsy	Microscopy (HE, PAS, Groccot)	Multifocal nodular lesions	Adiaspores were surrounded by a severe granulomatous inflammation with high number of epithelioid macrophages	600-800	Free ranging	100% (1/1)	58
2003-2005	UK	*Mus musculus*	*Emmonsia crescens*	Lung	Necropsy	Microscopy (HE, PAS, Groccot), Culture (Sabouraud's Glucose agar) Molecular (ITS1)	Multifocal nodular lesions	Adiaspores were surrounded by a granulomatous inflammation	NI	Free ranging	50% (2/4)	59
2003-2005	UK	*Rattus norvegicus*	*Emmonsia crescens*	Lung	Necropsy	Microscopy (HE, PAS, Groccot), Culture (Sabouraud's Glucose agar) Molecular (ITS1)	Multifocal nodular lesions	Adiaspores were surrounded by a granulomatous inflammation	NI	Free ranging	50% (1/2)	59
1967	USA	*Citella lateralis*	*Emmonsia parva*	Lung	Necropsy	Microscopy (HE, PAS, Groccot), culture (sabouraud's Glucose agar)	Multifocal nodular lesions	Adiaspores were surrounded by a granulomatous inflammation	10-25, 250-400	Free ranging	66.7% (4/6)	60
1967	USA	*Dipomys ordii*	*Emmonsia parva*	Lung	Necropsy	Microscopy (HE, PAS, Groccot), culture (sabouraud's Glucose agar)	Multifocal nodular lesions	Adiaspores were surrounded by a granulomatous inflammation	10-25, 250-400	Free ranging	15.5% (16/103	60
1967	USA	*Dipomys spectabolis*	*Emmonsia parva*	Lung	Necropsy	Microscopy (HE, PAS, Groccot), culture (sabouraud's Glucose agar)	Multifocal nodular lesions	Adiaspores were surrounded by a granulomatous inflammation	10-25, 250-400	Free ranging	100% (3/3)	60
1967	USA	*Eutamias minimus*	*Emmonsia parva*	Lung	Necropsy	Microscopy (HE, PAS, Groccot), culture (sabouraud's Glucose agar)	Multifocal nodular lesions	Adiaspores were surrounded by a granulomatous inflammation	10-25, 250-400	Free ranging	20% (1/5)	60
1967	USA	*Neotoma albigula*	*Emmonsia parva*	Lung	Necropsy	Microscopy (HE, PAS, Groccot), culture (sabouraud's Glucose agar)	Multifocal nodular lesions	Adiaspores were surrounded by a granulomatous inflammation	10-25, 250-400	Free ranging	25.8% (8/31)	60
1967	USA	*Onychomys leucogaster*	*Emmonsia parva*	Lung	Necropsy	Microscopy (HE, PAS, Groccot), culture (sabouraud's Glucose agar)	Multifocal nodular lesions	Adiaspores were surrounded by a granulomatous inflammation	10-25, 250-400	Free ranging	22.2% (2/9)	60
1967	USA	*Peromiscus maniculatus*	*Emmonsia parva*	Lung	Necropsy	Microscopy (HE, PAS, Groccot), culture (sabouraud's Glucose agar)	Multifocal nodular lesions	Adiaspores were surrounded by a granulomatous inflammation	10-25, 250-400	Free ranging	15.4% (2/13)	60
1967	USA	*Sigmodon hispidus*	*Emmonsia parva*	Lung	Necropsy	Microscopy (HE, PAS, Groccot), culture (sabouraud's Glucose agar)	Multifocal nodular lesions	Adiaspores were surrounded by a granulomatous inflammation	10-25, 250-400	Free ranging	30.4 (7/23)	60
1996	Zambia	*Fukomys anselli*	*Emmonsia parva var. crescens*	Lung	Necropsy	Microscopy (HE, PAS, Groccot), Culture (Sabhi agar)	Multifocal nodular lesions	Adiaspores were surrounded by a granulomatous inflammation	27-70	Free ranging	100% (20/20)	49
Carnivores												

1962	Argentina	*Conepatus chinga*	*Emmonsia sp.*	Lung	Necropsy	Microscopy (HE, PAS, Groccot)	Multifocal nodular lesions	Adiaspores were surrounded by a granulomatous inflammation	NI	Free ranging	12.5% (2/16)	33
1983	Canada	*Mephitis mephitis*	*Emmonsia crescens*	Lung	Necropsy	Microscopy (HE, PAS, Groccot), Electron microscopy	Multifocal nodular lesions	Adiaspores were surrounded by a granulomatous inflammation	60-150	Free ranging	28% (7/25)	61
1973	Czechoslovakia	*Vulpes vulpes*	*Emmonsia parva*	Lung	Necropsy	Microscopy (HE, PAS, Groccot), culture (sabouraud's Glucose agar)	Multifocal nodular lesions	Adiaspores were surrounded by a granulomatous inflammation	400-600	Free ranging	100% (1/1)	62
1971-1975	Czechoslovakia	*Alopex lagopus*	*Emmonsia crescens*	Lung	Necropsy	Microscopy (HE, PAS, Groccot), culture (sabouraud's Glucose agar)	Multifocal nodular lesions	Adiaspores were surrounded by a granulomatous inflammation	30-600	Free ranging	33.3% (1/3)	44
1971-1975	Czechoslovakia	*Lutra lutra*	*Emmonsia crescens*	Lung	Necropsy	Microscopy (HE, PAS, Groccot), culture (sabouraud's Glucose agar)	Multifocal nodular lesions	Adiaspores were surrounded by a granulomatous inflammation	30-600	Free ranging	100% (1/1)	44
1971-1975	Czechoslovakia	*Martes foina*	*Emmonsia crescens*	Lung	Necropsy	Microscopy (HE, PAS, Groccot), culture (sabouraud's Glucose agar)	Multifocal nodular lesions	Adiaspores were surrounded by a granulomatous inflammation	30-600	Free ranging	37.5% (6/16)	44
1971-1975	Czechoslovakia	*Martes martes*	*Emmonsia crescens*	Lung	Necropsy	Microscopy (HE, PAS, Groccot), culture (sabouraud's Glucose agar)	Multifocal nodular lesions	Adiaspores were surrounded by a granulomatous inflammation	30-600	Free ranging	72.2% (13/18)	44
1971-1975	Czechoslovakia	*Meles meles*	*Emmonsia crescens*	Lung	Necropsy	Microscopy (HE, PAS, Groccot), culture (sabouraud's Glucose agar)	Multifocal nodular lesions	Adiaspores were surrounded by a granulomatous inflammation	30-600	Free ranging	50% (2/4)	44
1971-1975	Czechoslovakia	*Mustela erminea*	*Emmonsia crescens*	Lung	Necropsy	Microscopy (HE, PAS, Groccot), culture (sabouraud's Glucose agar)	Multifocal nodular lesions	Adiaspores were surrounded by a granulomatous inflammation	30-600	Free ranging	39.5% (30/76)	44
1971-1975	Czechoslovakia	*Mustela nivalis*	*E. crescens (17), E. parva (1)*	Lung	Necropsy	Microscopy (HE, PAS, Groccot), culture (sabouraud's Glucose agar)	Multifocal nodular lesions	Adiaspores were surrounded by a granulomatous inflammation	30-600	Free ranging	33.9% (18/53)	44
1971-1975	Czechoslovakia	*Putorius eversmanni*	*E. crescens (19), E. parva (2)*	Lung	Necropsy	Microscopy (HE, PAS, Groccot), culture (sabouraud's Glucose agar)	Multifocal nodular lesions	Adiaspores were surrounded by a granulomatous inflammation	30-600	Free ranging	76.9% (21/26)	44
1971-1975	Czechoslovakia	*Putorius putorius*	*Emmonsia crescens*	Lung	Necropsy	Microscopy (HE, PAS, Groccot), culture (sabouraud's Glucose agar)	Multifocal nodular lesions	Adiaspores were surrounded by a granulomatous inflammation	30-600	Free ranging	30.6% (22/72)	44
1971-1975	Czechoslovakia	*Vulpes vulpes*	*E. crescens (3), E. parva (1)*	Lung	Necropsy	Microscopy (HE, PAS, Groccot), culture (sabouraud's Glucose agar)	Multifocal nodular lesions	Adiaspores were surrounded by a granulomatous inflammation	30-600	Free ranging	10.8% (4/37	44
1972-1975	Czechoslovakia	*Meles meles*	*Emmonsia parva var. crescens*	Lung	Necropsy	Microscopy (HE, PAS, Groccot), culture (sabouraud's Glucose agar)	Multifocal nodular lesions	Adiaspores were surrounded by a granulomatous inflammation	45-120	Free ranging	50% (2/4)	63
1972-1975	Czechoslovakia	*Lutra lutra*	*Emmonsia parva var. crescens*	Lung	Necropsy	Microscopy (HE, PAS, Groccot), culture (sabouraud's Glucose agar)	Multifocal nodular lesions	Adiaspores were surrounded by a granulomatous inflammation	45-300	Free ranging	100% (1/1)	63
1972-1975	Czechoslovakia	*Vulpes vulpes*	*Emmonsia parva var. crescens*	Lung	Necropsy	Microscopy (HE, PAS, Groccot), culture (sabouraud's Glucose agar)	Multifocal nodular lesions	Adiaspores were surrounded by a granulomatous inflammation	95-170	Free ranging	7% (3/43)	63
1999-2014	England	*Mustela sp*	*Emmonsia parva*	Lung	Necropsy	Microscopy (HE, PAS, Groccot)	Multifocal nodular lesions	Adiaspores were surrounded by a granulomatous inflammation	161	Free ranging	36% (4/11)	64
1999-2014	England	*Mustela erminea*	*Emmonsia parva*	Lung	Necropsy	Microscopy (HE, PAS, Groccot)	Multifocal nodular lesions	Adiaspores were surrounded by a granulomatous inflammation	202	Free ranging	60% (6/10)	64
1999-2014	England	*Mustela sp*	*Emmonsia parva*	Lung	Necropsy	Microscopy (HE, PAS, Groccot)	Multifocal nodular lesions	Adiaspores were surrounded by a granulomatous inflammation	205	Free ranging	29% (2/7)	64
2009	Italy	*Lutra lutra*	*Emmonsia sp.*	Lung	Necropsy	Microscopy (HE, PAS, Groccot)	No gross lesions	Adiaspores were surrounded by a severe granulomatous inflammation with high number of epithelioid macrophages	250	Free ranging	100% (1/1)	65
1998	UK	*Lutra lutra*	*Emmonsia parva*	Lung	Necropsy	Microscopy (HE, PAS, Groccot)	Multifocal nodular lesions	Adiaspores were surrounded by a granulomatous inflammation	240	Free ranging	100% (1/1)	66
2003-2005	UK	*Martes martes*	*Emmonsia crescens*	Lung	Necropsy	Microscopy (HE, PAS, Groccot), Culture (Sabouraud's Glucose agar) Molecular (ITS1)	Multifocal nodular lesions	Adiaspores were surrounded by a granulomatous inflammation	35	Free ranging	50% (1/2)	59
2003-2005	UK	*Mustela erminea*	*Emmonsia crescens*	Lung	Necropsy	Microscopy (HE, PAS, Groccot), Culture (Sabouraud's Glucose agar) Molecular (ITS1)	Multifocal nodular lesions	Adiaspores were surrounded by a granulomatous inflammation	10-110	Free ranging	28.6% (2/7)	59
2003-2005	UK	*Lutra lutra*	*Emmonsia crescens*	Lung	Necropsy	Microscopy (HE, PAS, Groccot), Culture (Sabouraud's Glucose agar) Molecular (ITS1)	Multifocal nodular lesions	Adiaspores were surrounded by a granulomatous inflammation	10-400	Free ranging	34.5% (19/35)	59
2003-2005	UK	*Vulpes vulpes*	*Emmonsia crescens*	Lung	Necropsy	Microscopy (HE, PAS, Groccot), Culture (Sabouraud's Glucose agar) Molecular (ITS1)	Multifocal nodular lesions	Adiaspores were surrounded by a granulomatous inflammation	48-70	Free ranging	14.3% (1/7)	59
2003-2005	UK	*Mustela nivalis*	*Emmonsia crescens*	Lung	Necropsy	Microscopy (HE, PAS, Groccot), Culture (Sabouraud's Glucose agar) Molecular (ITS1)	Multifocal nodular lesions	Adiaspores were surrounded by a granulomatous inflammation	68-85	Free ranging	20% (2/10)	59
Eulipotyphla												
1960	Bulgary	*Crocidura leucodon*	*Emmonsia crescens*	Lung	Necropsy	Microscopy (HE, PAS, Groccot), culture (sabouraud's Glucose agar)	Multifocal nodular lesions	Adiaspores were surrounded by a granulomatous inflammation	NI	Free ranging	0.6% (1/174)	35
1960	Bulgary	*Crocidura suaveolens*	*Emmonsia crescens*	Lung	Necropsy	Microscopy (HE, PAS, Groccot), culture (sabouraud's Glucose agar)	Multifocal nodular lesions	Adiaspores were surrounded by a granulomatous inflammation	NI	Free ranging	0.7% (2/298)	35
1960	Bulgary	*Erinaceus roumanicus*	*Emmonsia crescens*	Lung	Necropsy	Microscopy (HE, PAS, Groccot), culture (sabouraud's Glucose agar)	Multifocal nodular lesions	Adiaspores were surrounded by a granulomatous inflammation	NI	Free ranging	4.2% (1/24)	35
1960	Bulgary	*Sorex minutus*	*Emmonsia crescens*	Lung	Necropsy	Microscopy (HE, PAS, Groccot), culture (sabouraud's Glucose agar)	Multifocal nodular lesions	Adiaspores were surrounded by a granulomatous inflammation	NI	Free ranging	2.3% (5/218)	35
1960	Bulgary	*Talpa europaea*	*Emmonsia crescens*	Lung	Necropsy	Microscopy (HE, PAS, Groccot), culture (sabouraud's Glucose agar)	Multifocal nodular lesions	Adiaspores were surrounded by a granulomatous inflammation	NI	Free ranging	12.5% (1/8)	35
1957	Canada	*Sorex sp.*	*Emmonsia sp.*	Lung	Necropsy	Microscopy (HE)		Adiaspores were surrounded by a granulomatous inflammation	NI	Free ranging	100% (1/1)	67
1974	Czechoslovakia	*Some species (Sorex, Erinaceus,*	*Emmonsia crescens*	Lung	Necropsy	Microscopy (HE, PAS, Groccot)		Adiaspores were surrounded by a granulomatous inflammation	100-300	Free ranging	100% (69/69)	68

		Erinaceus, Talpa)										
1986	Czechoslovakia	*Sorex anareus*	*Emmonsia crescens*	Lung	Necropsy	Microscopy (HE, PAS, Groccot), Culture (Sabhi agar)	Multifocal nodular lesions	Adiaspores were surrounded by a granulomatous inflammation	225-634	Free ranging	0.9% (1/106)	40
1971-1975	Czechoslovakia	*Sorex araneus*	*Emmonsia crescens*	Lung	Necropsy	Microscopy (HE, PAS, Groccot), culture (sabouraud's Glucose agar)	Multifocal nodular lesions	Adiaspores were surrounded by a granulomatous inflammation	30-600	Free ranging	4% (6/150)	63
1971-1975	Czechoslovakia	*Talpa europaea*	*Emmonsia crescens*	Lung	Necropsy	Microscopy (HE, PAS, Groccot), culture (sabouraud's Glucose agar)	Multifocal nodular lesions	Adiaspores were surrounded by a granulomatous inflammation	30-600	Free ranging	16.7% (1/6)	63
1986-1997	Czechoslovakia	*Crocidura suaveolens*	*Emmonsia crescens*	Lung	Necropsy	Microscopy (HE, PAS, Groccot), Culture (Sabhi agar)	Multifocal nodular lesions	Adiaspores were surrounded by a granulomatous inflammation	46-451	Free ranging	5% (1/20)	45
1986-1997	Czechoslovakia	*Sorex araneus*	*Emmonsia crescens*	Lung	Necropsy	Microscopy (HE, PAS, Groccot), Culture (Sabhi agar)	Multifocal nodular lesions	Adiaspores were surrounded by a granulomatous inflammation	46-451	Free ranging	2% (13/529)	45
1988-1993	Czechoslovakia	*Crocidura suaveolens*	*Emmonsia parva var. crescens*	Lung	Necropsy	Microscopy (HE, PAS, Groccot), Culture (Sabhi agar)	Multifocal nodular lesions	Adiaspores were surrounded by a granulomatous inflammation	NI	Free ranging	20% (1/5)	40
1988-1993	Czechoslovakia	*Sorex anareus*	*Emmonsia parva var. crescens*	Lung	Necropsy	Microscopy (HE, PAS, Groccot), Culture (Sabhi agar)	Multifocal nodular lesions	Adiaspores were surrounded by a granulomatous inflammation	NI	Free ranging	11.8% (2/17)	40
1968	Nepal	*Soriculus nigrescens*	*Emmonsia crescens*	Lung	Necropsy	Microscopy (HE, PAS, Groccot)	Multifocal nodular lesions	Adiaspores were surrounded by a granulomatous inflammation	300-500	Free ranging	2.3% (1/44)	51
2006	Portugal	*Erinaceus europaeus*	*Chrysosporium parvum var crescens*	Lung	Necropsy	Microscopy (HE, PAS, Groccot)	Multifocal nodular lesions	Adiaspores were surrounded by a granulomatous inflammation	119-285	Free ranging	100% (1/1)	69
2003-2005	UK	*Talpa europaea*	*Emmonsia crescens*	Lung	Necropsy	Microscopy (HE, PAS, Groccot), Culture (Sabouraud's Glucose agar) Molecular (ITS1)	Multifocal nodular lesions	Adiaspores were surrounded by a granulomatous inflammation	28	Free ranging	33.3% (1/3)	59
Didelphimorphids												
1982	Australia	*Lasiorhinus latifrons*	*Emmonsia parva*	Lung	Necropsy	Microscopy (HE, PAS, Groccot), culture (sabouraud's Glucose agar)	No gross lesions	Adiaspores were surrounded by a granulomatous inflammation	22	Free ranging	100% (6/6)	70
2017	Australia	*Lasiorhinus krefftii*	*Emmonsia parvum var. Crescens*	Lung	Necropsy	Microscopy (HE, PAS, Groccot)	Interstitial fibrosis	Adiaspores were surrounded by a granulomatous inflammation	15-22	Free ranging	100% (2/2)	71
1966	New Zealand	*Trichosurus vulpecula*	*Emmonsia sp.*	Lung	Necropsy	Microscopy (HE, PAS, Groccot), culture (sabouraud's Glucose agar)	Multifocal nodular lesions	Adiaspores were surrounded by a granulomatous inflammation	300-420	Free ranging	100% (1/1)	72
1992	New Zealand	*Trichosurus vulpecula*	*Emmonsia crescens*	Lung	Necropsy	Microscopy (HE, PAS, Groccot)	Multifocal subpleural granulomas	Adiaspores were surrounded by a granulomatous inflammation	250-340	Free ranging	88.2% (15/17)	73
Cingulata												
1962	Argentina	*Chaetophractus villosus*	*Emmonsia sp.*	Lung	Necropsy	Microscopy (HE, PAS, Groccot)	Multifocal nodular lesions	Adiaspores were surrounded by a granulomatous inflammation	NI	Free ranging	90% (9/10)	74
1962	Argentina	*Dasypus septemcinctus*	*Emmonsia sp.*	Lung	Necropsy	Microscopy (HE, PAS, Groccot)	Multifocal nodular lesions	Adiaspores were surrounded by a granulomatous inflammation	NI	Free ranging	100% (1/1)	74
2010	Brazil	*Dasypus novemcinctus*	*Emmonsia parvum var. Crescens*	Lung	Necropsy	Molecular (ITS4, ITS5)			NI	Free ranging	50% (1/2)	76
Lagomorphs												
1960	Bulgary	*Lepus europaeus*	*Emmonsia crescens*	Lung	Necropsy	Microscopy (HE, PAS, Groccot), culture (sabouraud's Glucose agar)	Multifocal nodular lesions	Adiaspores were surrounded by a granulomatous inflammation	NI	Free ranging	2.7% (1/37)	35
2018	UK	*Oryctolagus cuniculus*	*Emmonsia sp.*	Lung	Necropsy	Microscopy (HE, PAS, Groccot), Culture (Sabhi agar)	Multifocal nodular lesions	Adiaspores were surrounded by a granulomatous inflammation	190-290	Free ranging	100% (1/1)	76
1967	USA	*Sylvilagus audubonii*	*Emmonsia parva*	Lung	Necropsy	Microscopy (HE, PAS, Groccot), culture (sabouraud's Glucose agar)	Multifocal nodular lesions	Adiaspores were surrounded by a granulomatous inflammation	10-25, 250-400	Free ranging	7.1% (1/14)	60
Artiodactyla												
2014	Hokaido, Japan	*Cervus nippon yesoensis*	*Emmonsia crescens*	Lung	Necropsy	Microscopy (HE, PAS, Groccot)	No gross lesions	Adiaspores were surrounded by a granulomatous inflammation	227-260	Free ranging	100% (1/1)	77
Perissodactyla												
2002	USA	*Equus caballus*	*Emmonsia crescens*	Lung	Biopsy	Microscopy (HE, PAS, Groccot), molecular (28S)	Multifocal nodular lesions	Adiaspores were surrounded by a granulomatous inflammation	30-80	-	100% (1/1)	78
Amphibia												
Anura												
1996	USA	*Rana catesbeiana*	*Emmonsia parva var. crescens*	Skeletal muscle	Necropsy	Microscopy (HE, PAS, Groccot)	Multifocal nodular lesions	Adiaspores were surrounded by a granulomatous inflammation	240	Captive	100% (1/1)	79

DFA=Diameter of Fungal adiaspores; NI=No information

References

1. Fielli, M., Ceccato, A., Capece, P., Posse, G., Monteverde, A., Gonzalez, A. Human Adiaspiromycosis: A Case From Argentina. *Chest*, **144(4)**, 220A (2013).
2. Moraes, M. A., Almeida, M. C. D., Raick, A. N. Caso fatal de adiaspiromicose pulmonar humana. *Rev. Inst. Med. Trop. São Paulo*, **31(3)**, 188-194 (1989).
3. Barbas Filho, J. V., Amnto, M. B. P., Deheinzelin, D., Saldiva, P. H. N., de Carvalho, C. R. R. Respiratory failure caused by adiaspiromycosis. *Chest*, **97(5)**, 1171-1175 (1990).
4. Peres, L. C., Figueiredo, F., Peinado, M., & Soares, F. A. Fulminant disseminated pulmonary adiaspiromycosis in humans. *Am. J. Trop. Med. Hyg*., **46(2)**, 146-150 (1992).
5. de Almeida Barbosa, A., Moreira Lemos, A. C., Severo, L. C. Acute pulmonary adiaspiromycosis. Report of three cases and review of 16 other cases collected from the literature. *Rev. Iberoam. Micol.*, **14**, 177-180 (1997).
6. Martins, R. L. M., Santos, C. G., França, F. R. F., Moraes, M. A. Adiaspiromicose humana. Relato de um caso tratado com cetoconazol. *Rev. Soc. Bras. Med. Trop.*, **30(6)**, 507-509 (1997).
7. Lima, T. S. M., Moraes, M. A., Magalhães, H. Q., Athayde, N. S. G. Novo caso de adiaspiromicose humana diagnosticado por biópsia transbrônquica. *J. Pneumologia*, **24**, 339-341 (1998).
8. Santos, V. M. D., Fatureto, M. C., Saldanha, J. C., Adad, S. J. Pulmonary adiaspiromycosis: report of two cases. *Rev. Soc. Bra. Med. Trop.*, **33(5)**, 483-488 (2000).
9. Moraes, M. A., Gomes, M. I. Adiaspiromicose humana: lesões cicatriciais em linfonodos do mediastino. *Rev. Soc. Bra. Med. Trop*., **37(2)**, 177-178 (2004).
10. Mendes, M. O., Moraes, M. A., Renoiner, E. I., Dantas, M. H., Lanzieri, T. M., Fonseca, C. F., Hatch, D. L. Acute conjunctivitis with episcleritis and anterior uveitis linked to adiaspiromycosis and freshwater sponges, Amazon region, Brazil, 2005. *Emerg. Infect. Dis.*, **15(4)**, 633 (2009).
11. Silva, R. M., Liporoni, G. A., Botto, C. C., Rodrigues, B. C., Scudeler, D., Cunha Junior, W. Adiaspiromicose pulmonar tratada sem antifúngicos. *Rev. Soc. Bras. Med. Trop*., **43(1),** 95-97 (2010).
12. Santos, L. G. D., Araújo, J. K. L., Tavares, A. C. B., Fé, J. D. M. D. A. Adiaspiromicose pulmonar humana. *JBPML*, **45(4)**, 313-316 (2009).
13. Feng, P., Yin, S., Zhu, G., Li, M., Wu, B., Xie, Y., Lu, C. Disseminated infection caused by *Emmonsia pasteuriana* in a renal transplant recipient. *Int. J. Dermatol.*, **42(12),** 1179-1182 (2015).
14. KoĎousek, R., Vortel, V., Fingerland, A., Vojtek, V., Šerý, Z., Hajek, V., Kučera, K. Pulmonary adiaspiromycosis in man caused by *Emmonsia crescens*: report of a unique case. *Am. J. Clin. Pathol*., **56(3)**, 394-399 (1971).
15. Nuorva, K., Pitkänen, R., Issakainen, J., Huttunen, N. P., Juhola, M. Pulmonary adiaspiromycosis in a two year old girl. *J. Clin. Pathol.*, **50(1)**, 82-85 (1997).
16. Dot, J. M., Debourgogne, A., Champigneulle, J., Salles, Y., Brizion, M., Puyhardy, J. M., Machouart, M. Molecular diagnosis of disseminated adiaspiromycosis due to Emmonsia crescens. *J. Clin. Microbiol*., **47(4)**, 1269-1273 (2009).
17. Wellinghausen, N., Kern, W. V., Haase, G., Rozdzinski, E., Kern, P., Marre, R., Hetzel, M. Chronic granulomatous lung infection caused by the dimorphic fungus *Emmonsia* sp. *Int. J. Med. Microbiol.*, **293(6)**, 441-445 (2003).
18. Kamalam, A., Thambiah, A. S. Adiaspiromycosis of human skin caused by *Emmonsia crescens*. *J. Med. Vet. Mycol*., **17(4)**, 377-381 (1979).
19. Malik, R., Capoor, M. R., Vanidassane, I., Gogna, A., Singh, A., Sen, B., Chakrabarti, A. Disseminated *Emmonsia pasteuriana* infection in India: a case report and a review. *Mycoses*, **59(2)**, 127-132 (2016).
20. Turner, D., Burke, M., Bashe, E., Blinder, S., Yust, I. Pulmonary adiaspiromycosis in a patient with acquired immunodeficiency syndrome. *Eur. J. Clin. Microbiol. Infect. Dis.*, **18(12)**, 893-895 (1999).
21. Martin, M., Fernández, E., Bertoli, F., Pinilla, E. Adispiromicosis Humana: Primer Caso Panameño. *Ver. Méd. Cient*., **14(2)** (2001).
22. Lochan, H., Naicker, P., Maphanga, T., Ryan, A., Pillay, K., Govender, N. P., Eley, B. A case of emmonsiosis in an HIV-infected child. *S. Afr. J. HIV Med.*, **16(1)**, 1-4 (2015).
23. Kenyon, C., Bonorchis, K., Corcoran, C., Meintjes, G., Locketz, M., Lehloenya, R., Bamford, C. A dimorphic fungus causing disseminated infection in South Africa. *N. Engl. J. Med.*, **369(15)**, 1416-1424 (2013).
24. Pelegrín, I., Ayats, J., Xiol, X., Cuenca-Estrella, M., Jucglà, A., Boluda, S., Cabellos, C. Disseminated adiaspiromycosis: case report of a liver transplant patient with human immunodeficiency infection, and literature review. *Transpl. Infect. Dis*., **13(5)**, 507-514 (2011).
25. Buyuksirin, M., Ozkaya, S., Yucel, N., Guldaval, F., Ceylan, K., Polat, G. E. Pulmonary adiaspiromycosis: The first reported case in Turkey. *Respir. Med. CME*, **4(4)**, 166-169 (2011).
26. Denson, J. L., Keen, C. E., Froeschle, P. O., Toy, E. W., Borman, A. M. Adiaspiromycosis mimicking widespread malignancy in a patient with pulmonary adenocarcinoma. *J. Clin. Pathol.*, **62(9)**, 837-839 (2009).
27. Tang, X. H., Zhou, H., Zhang, X. Q., De Han, J., Gao, Q. Cutaneous disseminated emmonsiosis due to *Emmonsia pasteuriana* in a patient with cytomegalovirus enteritis. *JAMA Dermat*., **151(11)**, 1263-1264 (2015).
28. Anstead, G. M., Sutton, D. A., Graybill, J. R. Adiaspiromycosis causing respiratory failure and a review of human infections due to Emmonsia and *Chrysosporium* spp. J. Clin. Microbiol., **50(4)**, 1346-1354 (2012).
29. Stebbins, W. G., Krishtul, A., Bottone, E. J., Phelps, R., Cohen, S. Cutaneous adiaspiromycosis: a distinct dermatologic entity associated with *Chrysosporium* species. *JAAD*, **51(5)**, S185-S189 (2004).
30. Bottone, E. J. Histologic diagnosis of cutaneous adiaspiromycosis in the absence of successful culture of *Emmonsia crescens* or *Emmonsia parva*. *Clin. Microbiol. Newsl*., **17(33)**, 131-134 (2011).
31. Kappagoda, S., Adams, J. Y., Luo, R., Banaei, N., Concepcion, W., Ho, D. Y. Fatal *Emmonsia* sp. infection and fungemia after orthotopic liver transplantation. *Emerg. Infect. Dis*., **23(2)**, 346 (2017).
32. Salfelder, K., Fingerland, A., De Mendelovici, M. Two cases of adiaspiromycosis. *Beiträge zur Pathologie*, **148(1)**, 94-100 (1973).
33. Albassam, W. L., Lord, R. D. Adiaspiromycosis in Argentine mammals. *Mycologia*, **56(3)**, 374-383 (1964).
34. Hubálek, Z. Adiaspiromycosis of wild small mammals in Austria. *Fol. Parasitologica*, **26**, 159-164 (1979).
35. Zlatanov, Z., Genov, T. Isolation of *Emmonsia crescens* Emmons et Jellison 1960 from small mammals in Bulgaria. *Mycopathologia*, **56(1)**, 1-3 (1975).
36. Bakerspigel, A. Canadian species of *Sorex*, *Microtus* and *Peromyscus* infected with *Emmonsia*. *Mycopathologia*, **34(3-4),** 273-279 (1968).
37. Tobon, J. L., Yuill, T. M., Samuel, W. M. Adiaspiromycosis in the Franklin's ground squirrel, *Spermophilus franklini*, and pika, *Ochotona princeps*, from Alberta, Canada. *J. Wildl. Dis*., **12(1)**, 97-100 (1976).
38. Leighton, F. A., Wobeser, G. The prevalence of adiaspiromycosis in three sympatric species of ground squirrels. *J. Wild. Dis.*, **14(3)**, 362-365 (1978).
39. Křivanec, K., Otčenášek, M., Prokopič, J. Experimental adiaspiromycosis of the common vole (*Microtus arvalis*) and other small wild mammals after intraperitoneal inoculation. *Mycopathologia*. **53(1-4)**, 133-140 (1974).
40. Hubalek, Z., Juricova, Z., Zima, J. Adiaspiromycosis of mammals in an air-polluted area of Czechoslovakia. *Ekologia CSSR*, **7(3)**, 281-289 (1988).
41. Hubálek, Z., Zejda, J., Nesvadbová, J., Rychnovský, B. Adiasporomycosis - A widespread disesase. *Folia Zool.*, **40(2)**, 107-116 (1991).
42. Hubálek, Z., Zejda, J., Svobodová, Š., Kučera, J. Seasonality of rodent adiasporomycosis in a lowland forest. *J. Med. Vet. Mycol.*, **31(5)**, 359-366 (1993).
43. Hubalek, Z., Nesvadbova, J., Halouzka, J. Emmonsiosis of rodents in an agroecosystem. *Med. Mycol. J*., **36(6)**, 387-390 (1998).
44. Křivanec, K. Adiaspiromycosis in Czechoslovakian mammals. *J. Med. Vet. Mycol*., **15(3)**, 221-223 (1977).
45. Hubálek, Z.Emmonsiosis of wild rodents and insectivores in Czechland. *J. Wildl Dis*., **35(2)**, 243-249 (1999).
46. Hubálek, Z., Gaisler, J., Nesvadbová, J. Emmonsiosis of small mammals (*Rodentia*, *Insectivora*) in the Pálava Biosphere Reserve of the UNESCO. *Acta Soc. Zool. Bohemoslov*, **62**, 101-104 (1998).
47. Hubálek, Z., Nesvadbová, J., Rychnovsky, B. A heterogeneous distribution of *Emmonsia parva* var. *crescens* in an agro-ecosystem. *J. Med. Vet. Mycol.*, **33(3)**, 197-200 (1995).
48. Fischer, O. A. Adiaspores of *Emmonsia parva* var. *crescens* in lungs of small rodents in a rural area. *Acta Vet. Brno*, **70(3)**, 345-352 (2001).
49. Hubálek, Z., Burda, H., Scharff, A., Heth, G., Nevo, E., Šumbera, R., Zima, J. Emmonsiosis of subterranean rodents (*Bathyergidae*, *Spalacidae*) in Africa and Israel. *Med. Mycol. J*., **43(8)**, 691-697 (2005).
50. Morandi, F., Galuppi, R., Buitrago, M. J., Delogu, M., Lowenstine, L. J., Panarese, S., Sarli, G. Disseminated pulmonary adiaspiromycosis in a crested porcupine (*Hystrix cristata* Linnaeus, 1758). *J. Wildl. Dis*., **48(2)**, 523-525 (2012).
51. Ohbayashi, M., Ishimoto, Y. Two cases of adiaspiromycosis in small mammals. *Jap. J. Vet. Res*., **19(4)**, 103-105 (1971).
52. 谷山弘行, 古岡秀文, 松井高峯, & 小野威. Two cases of adiaspiromycosis in the Japanese pika (*Ochotona hyperborea yesoensis Kishida*). *Jap. J. Vet. Sci.*, **47(1)**, 139-142 (1985).
53. 中野美絵, 山口英美, 木元美樹, 崔宝隠, 松本高太郎, 豊留孝仁, & 古岡秀文. 北海道十勝地方のエゾリス (*Sciurus vulgaris orientis*) および ドブネズミ (*Rattus norvegicus*) にみられた adiaspiromycosis の病理学的検索. *日本野生動物医学会誌*, **22(2)**, 37-40 (2017).
54. Kim, T. H., Han, J. H., Chang, S. N., Kim, D. S., Abdelkader, T. S., Seok, S. H., Shin, J. H. Adiaspiromycosis of an *Apodemus agrarius* captured wild rodent in Korea. *Lab. Anim. Res*., **28(1)**, 67-69 (2012).
55. Dolka, I., Gizejewska, A., Gizejewski, Z., Kolodziejska-Lesisz, J., Klucinski, W. Pulmonary adiaspiromycosis in the Eurasian beaver (*Castor fiber*) inhabiting Poland. *Pol. J. Vet. Sci.*, **20(3)** (2017).
56. Paldrok, H., Zetterberg, B. A contribution to the study on the occurrence of adiaspiromycosis (haplomycosis) in rodents in Sweden. *Acta Pathol. Microbiol. Scand*, **56(1)**, 65-69 (1962).

57. Mörner, T., Avenäs, A., Mattsson, R. Adiaspiromycosis in a European beaver from Sweden. *J. Wildl. Dis*., **35(2)**, 367-370 (1999).
58. Chantrey, J. C., Borman, A. M., Johnson, E. M., Kipar, A. *Emmonsia crescens* infection in a British water vole (*Arvicola terrestris*). *Sabouraudia*, **44(4)**, 375-378 (2006).
59. Borman, A. M., Simpson, V. R., Palmer, M. D., Linton, C. J., Johnson, E. M. Adiaspiromycosis due to *Emmonsia crescens* is widespread in native British mammals. *Mycopathologia,* **168(4),** 153-163 (2009).
60. Taylor, R. L., Miller, B. E., Rust Jr, J. H. Adiaspiromycosis in small mammals of New Mexico. *Mycologia*, **59(3)**, 513-518 (1967).
61. Albassam, M. A., Bhatnagar, R., Lillie, L. E., Roy, L. Adiaspiromycosis in striped skunks in Alberta, Canada. *J. Wildl. Dis.*, **22(1)**, 13-18 (1986).
62. Otčenásek, M., Krivanec, K., Slais, J. *Emmonsia parva* as causal agent of adiaspiromycosis in a fox. *Sabouraudia*, **13(1)**, 52-57 (1975).
63. Křivanec, K., Otčenášek, M., Šlais, J. Adiaspiromycosis in large free-living carnivores. *Mycopathologia*, **58(1)**, 21-25 (1976).
64. Simpson, V. R., Tomlinson, A. J., Stevenson, K., McLuckie, J. A., Benavides, J., Dagleish, M. P. A post-mortem study of respiratory disease in small mustelids in south-west England. *BMC Vet. Res*., **12(1)**, 72 (2016).
65. Malatesta, D., Simpson, V. R., Fontanesi, L., Fusillo, R., Marcelli, M., Bongiovanni, L., Della Salda, L. First description of adiaspiromycosis in an Eurasian otter (*Lutra lutra*) in Italy. *Vet. Ital*., **50(3)**, 199-202 (2014).
66. Simpson, V. R., Gavier-Widen, D. Fatal adiaspiromycosis in a wild Eurasian otter (*Lutra lutra*). *Vet. Rec*., **147(9)**, 239-241 (2000).
67. Bakerspigel, A. Haplomycosis (adiaspiromycosis) in *Sorex*. *Can. J. Microbiol*, **7(4),** 676-676 (1961).
68. Blažek, K., Prokopič, J. Contribution to the pathology of spontaneous adiaspiromycosis in small mammals. *Ann. Parasitol. Hum. Comp*., **49(2),** 229-239 (1974).
69. Seixas, F., Travassos, P., Pinto, M. L., Pires, I., Pires, M. A. Pulmonary adiaspiromycosis in a European hedgehog (*Erinaceus europaeus*) in Portugal. *Vet. Rec.*, **158**: 274-275 (2006).
70. Mason, R. W., Gauhwin, M. Adiaspiromycosis in south Australian hairy-nosed wombats (*Lasiorhinus latifrons*). *J. Wildl. Dis*., **18(1)**, 3-8 (1982).
71. Schaffer-White, A. B., Harper, D., Mayhew, M., McKinnon, A., Knott, L., Allavena, R. E. Pulmonary adiaspiromycosis in critically endangered northern hairy-nosed wombats (*Lasiorhinus krefftii*). *Aust. Vet. J.*, **95(11)**, 431-436 (2017).
72. Smith, J. M. B., Lancaster, M. C. Adiaspiromycosis in the brush opossum, *Trichosurus vulpecula*, in New Zealand. *J. Med. Vet. Mycol.*, **4(3)**, 146-147 (1966).
73. Johnstone, A. C., Hussein, H. M., Woodgyer, A. Adiaspiromycosis in suspected cases of pulmonary tuberculosis in the common brushtail possum *Trichosurus vulpecula*. *N. Z. Vet. J*., **41(4)**, 175-178 (1993).
74. Jellison, W. L., Lord, R. D. Adiaspiromycosis in Argentine mammals. *Mycologia,* **56(3)**, 374-383 (1964).
75. Richini-Pereira, V. B., Bosco, S. M. G., Theodoro, R. C., Barrozo, L., Bagagli, E. Road-killed wild animals: a preservation problem useful for eco-epidemiological studies of pathogens. *J. Venom. Anim. Toxins incl. Trop. Dis*., **16(4)**, 607-613 (2010).
76. Hughes, K., Borman, A. M. Adiaspiromycosis in a wild European rabbit, and a review of the literature. *JVDI*, **30(4)**, 614-618 (2018).
77. Matsuda, K., Niki, H., Yukawa, A., Yanagi, M., Souma, K., Masuko, T., Taniyama, H. First detection of adiaspiromycosis in the lungs of a deer. *J. Vet. Med. Sci.*, **77(8)**, 981-983 (2015).
78. Pusterla, N., Pesavento, P. A., Leutenegger, C. M., Hay, J., Lowenstine, L. J., Durando, M. M., Magdesian, K. G. Disseminated pulmonary adiaspiromycosis caused by Emmonsia crescens in a horse. *Equine Vet. J*., **34(7)**, 749-752 (2002).

Hill, J. E., Parnell, P. G. Adiaspiromycosis in bullfrogs (*Rana catesbeiana*). *JVDI*, **8(4),** 496-497 (1996).

Supplementary Figure 1. Gross findings associated to Motor Vehicle Collisions in Armadillos.

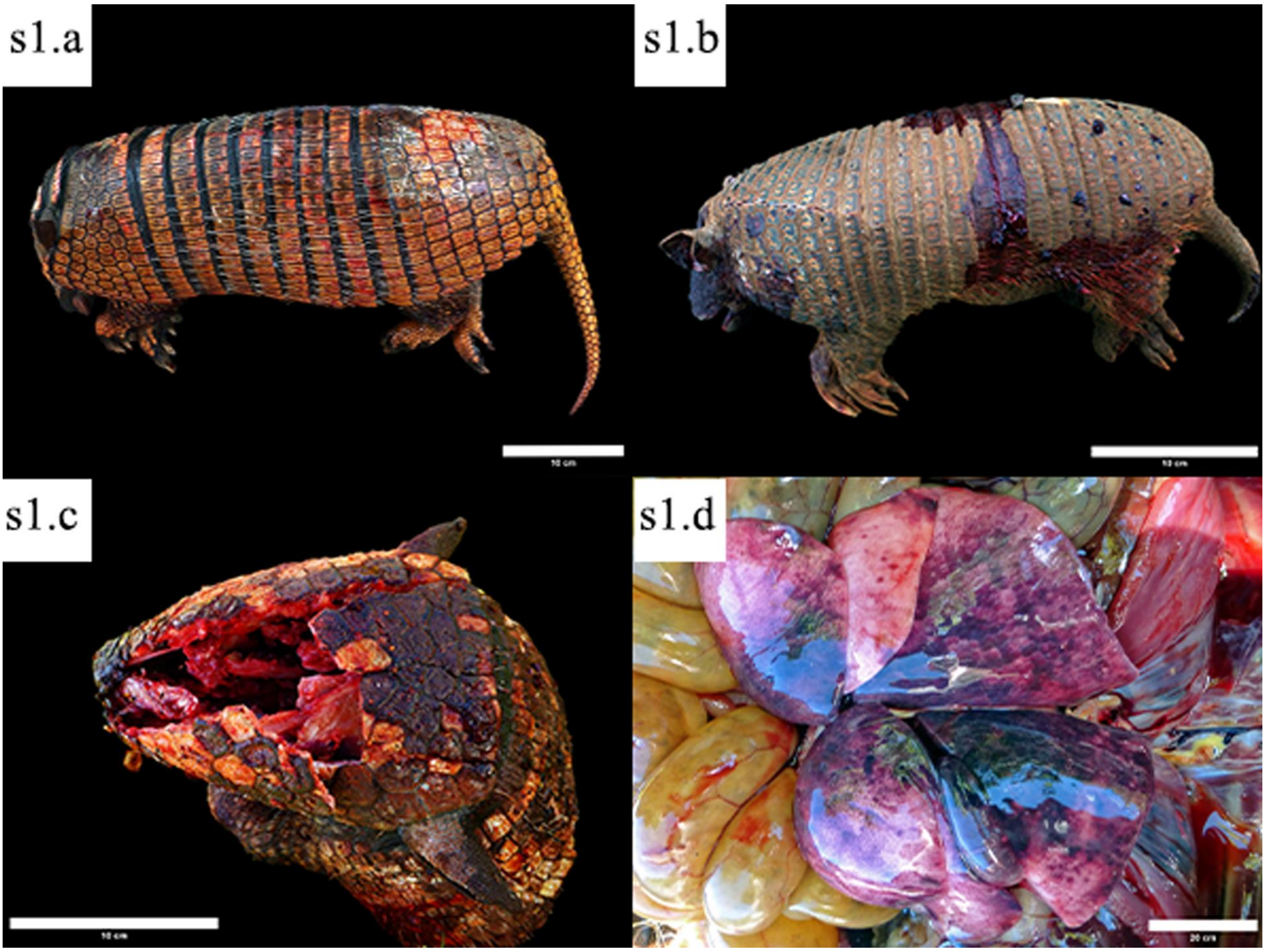

LEGENDS

Supplementary Figure 1. S1.A) Case 10; adult, female *Euphractus sexcinctus*. Whole body, left lateral view. S1.B) Case 4; adult, male *Cabassous unicinctus*. Note linear rupture (arrow) of the carapace at the left lateral aspect of the cervical and shoulder regions, as well as the presence of multifocal hemorrhage coating the carapace. S1.C) Case 11; adult, male *E. sexcinctus*. Note severe cranioencephalic trauma. S1.D) Case 7; adult, female *E. sexcinctus*. All lung lobes failed to collapse and have multifocal, poorly demarcated dark red foci.
